# Fragment-derived modulators of an industrial β-glucosidase

**DOI:** 10.1042/BCJ20200507

**Published:** 2020-11-26

**Authors:** Eleni Makraki, John F. Darby, Marta G. Carneiro, James D. Firth, Alex Heyam, Eiso Ab, Peter O'Brien, Gregg Siegal, Roderick E. Hubbard

**Affiliations:** 1 Department of Chemistry, University of York, Heslington, York YO10 5DD, U.K.; 2 Department of Infection, Immunity and Cardiovascular Disease, University of Sheffield Medical School, Beech Hill, Sheffield, U.K.; 3 ZoBio BV, J.H. Oortweg 19, 2333 CH Leiden, The Netherlands; 4 Division of Medicinal Chemistry, Amsterdam Institute for Molecules, Medicines and Systems (AIMMS), Vrije Universiteit, De Boelelaan 1108, 1081 HZ Amsterdam, Netherlands; 5 Vernalis Research, Granta Park, Abington, Cambridge CB21 6GB, U.K.

**Keywords:** glycoside hydrolase, NMR spectroscopy, protein–ligand docking, small molecule activators, *Tr*Bgl2

## Abstract

A fragment screen of a library of 560 commercially available fragments using a kinetic assay identified a small molecule that increased the activity of the fungal glycoside hydrolase *Tr*Bgl2. An analogue by catalogue approach and detailed kinetic analysis identified improved compounds that behaved as nonessential activators with up to a 2-fold increase in maximum activation. The compounds did not activate the related bacterial glycoside hydrolase *Cc*BglA demonstrating specificity. Interestingly, an analogue of the initial fragment inhibits both *Tr*Bgl2 and *Cc*BglA, apparently through a mixed-model mechanism. Although it was not possible to determine crystal structures of activator binding to 55 kDa *Tr*Bgl2, solution NMR experiments demonstrated a specific binding site for the activator. A partial assignment of the NMR spectrum gave the identity of the amino acids at this site, allowing a model for *Tr*Bgl2 activation to be built. The activator binds at the entrance of the substrate-binding site, generating a productive conformation for the enzyme–substrate complex.

## Introduction

The depletion of fossil fuel in combination with the increasing demand for energy worldwide has stimulated research on alternative and sustainable energy sources. β-glucosidases (EC 3.2.1.21), which hydrolyse β-1,4-glycosidic bonds, have received considerable attention due to their essential role in bioethanol production from lignocellulosic (LC) biomass such as wood, agricultural residues and dedicated energy crops [1]. LC biomass is composed mainly of cellulose (40–50%), in combination with hemicellulose (25–30%) and lignin (15–20%) [1]. A mixture of enzymes catalyses cellulose degradation and comprises three categories; endoglucanases (EC 3.2.1.4), exoglucanases or cellobiohydrolases (EC 3.2.1.91) and β-glucosidases (EC 3.2.1.21) [2,3]. Endoglucanases cleave the internal β-1,4-glycosidic bonds of cellulose releasing small fragments. Subsequently, exoglucanases or cellobiohydrolases (CBH) act on the reducing and non-reducing ends resulting in short-chain cello-oligosaccharides such as cellobiose, which are then hydrolysed into glucose by the action of β-glucosidases [2]. However, the low activity of β-glucosidase results in the accumulation of cellobiose and subsequent inhibition of other cellulases [4,5,6,7]. To counter these effects biomass hydrolysis requires high concentrations of β-glucosidases, increasing the cost and reducing the efficiency of large-scale conversions [6,7]. There is great interest therefore in identifying β-glucosidases with improved catalytic activity and thermostability.

**Figure 1.**
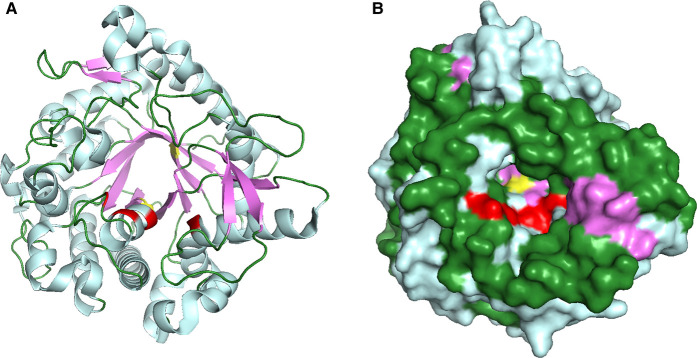
The crystal structure of TrBgl2. (**A**) Cartoon and (**B**) Molecular surface representation of the *Tr*Bgl2 structure (PDB ID 3AHY) [11]. The two catalytic glutamic acid residues of *Tr*Bgl2 are shown in yellow. The helices are shown in cyan, the strands in magenta and loops in green. The activity-enhanced mutants are shown in red. The figures were created in PyMOL.

The fungus *Trichoderma reesei* (*Tr*Bgl2) produces thermostable cellulolytic enzymes, which make them attractive targets for industrial applications [8]. The glycosyl hydrolase, *TrBgl*2 from this organism, belongs to the β-retaining glycoside hydrolase family 1 according to the classification of carbohydrate-active enzymes (CAZY) [9,10]. The structure and biochemical properties of *Tr*Bgl2 have been elucidated by Jeng et al. [11]. As shown in Figure 1, *Tr*Bgl2 adopts a (*α/β*)_8_-ΤΙΜ barrel-like fold as a typical member of the GH1 enzyme family [11]. The active site of the enzyme is in a 15–20 Å deep slot-like cleft and is surrounded by negatively charged residues. The catalytic acid/proton donor is Glu165 (E165) while the catalytic nucleophile/base is Glu367 (E367) for *Tr*Bgl2 [11]. Mutation of amino acids in the substrate entrance region away from the active site such as P172L, L167W, T178V and P172L/F250A gave enhanced *k*_cat_/*K*_m_ values by 5.3-fold compared with the wild type (WT) [12]. Similarly, modified β-glucosidases from *T. reesei* are included in cellulolytic enzyme cocktails that have been commercialised.

Although the majority of applications of fragment-based ligand discovery (FBLD) approaches focus on enzyme inhibition, our group has previously described the identification of the first small molecule that directly activates a glucosidase, the bacterial *O*-GlcNAc hydrolase from *Bacteroides thetaiotaomicron* (BtGH84) [13]. This provided the experience to use FBLD methods to explore the activation of *Tr*Bgl2 by small molecules. The work reported here is the identification and validation of a novel activator of the fungal *Tr*Bgl2. Ligand-observed NMR and thermal shift assays were used to further characterise the binding of the activator to *Tr*Bgl2, whilst protein-observed NMR experiments combined with molecular docking were used to generate a model to give insight into the mechanism of small molecule activation of *Tr*Bgl2.

## Materials and methods

### Chemicals and reagents

The fragment library was assembled as described by Schulz et al. [14], dissolved in DMSO-d_6_ to a concentration of 200 mM and quality controlled using ^1^H NMR spectroscopy. The library is a subset of the Maybridge Ro3 diversity library (https://www.maybridge.com/portal/alias__Rainbow/lang__en/tabID__230/DesktopDefault.aspx). The entire library contains 2500 compounds of which 560 compounds were selected for maximum diversity in chemical representation and higher solubility. Additional neighbours were purchased from Chembridge and Enamine.

### Cloning

The gene *T. reesei* (*Trbgl2*; AB003110) was optimised to the favoured codon usage for *Escherichia coli* and synthesised by Invitrogen. The *Trbgl2* gene fragment was amplified by polymerase chain reaction (PCR) with forward 5′-CCAGGGACCAGCA ATGCTGCCGAAAGATTTTCAGTGG-3′ and reverse 5′-GAGGAGAAGGCGCGTTATGCTGCTGCAATCAGTTCATCAAAC-3′ primers (overhangs underlined). The PCR product was cloned into the expression vector of pET-YSBLIC3C using the NEBuilder HiFi DNA Assembly Cloning kit (Biolabs). The resulting DNA construct pET-YSBLIC3C_*Trbgl2* was sequenced and verified.

### Site-directed mutagenesis

The appropriate codon in the previously obtained recombinant plasmid pET-YSBLIC3C_*Trbgl2* was mutated by PCR in order to obtain the E367Q substitution in *Tr*Bgl2. The *E367Q Trbgl2* gene fragment was produced by PCR with forward 5′-CCTATTTATGTTACCCAAAATGGCACCAG-3′ and reverse 5′-GCCATTTTGGGTAACATAAATAGGCGGA-3′ primers (overhangs underlined). The PCR product was cloned into the expression vector of pET-YSBLIC3C using a NEBuilder HiFi DNA Assembly Cloning kit (Biolabs). The resulting DNA construct pET-YSBLIC3C_E367Q*Trbgl2* was sequenced and verified.

### *Tr*bgl2 and E367Q *Tr*Bgl2 expression and purification

#### Unlabelled protein

*Tr*Bgl2 and the mutant E367Q *Tr*Bgl2 were expressed and purified in an analogous manner to that previously described by Jeng et al. [11].

#### ^15^N Isotope-labelled *Tr*Bgl2 and E367Q *Tr*Bgl2 protein

Production of ^15^N-labelled *Tr*Bgl2 and E367Q *Tr*Bgl2 followed a similar protocol to the unlabelled proteins. An overnight *Tr*Bgl2/E367Q *Tr*Bgl2 LB medium preculture from a freshly transformed colony was pelleted by centrifugation at room temperature (4000 rpm, 10 min) and resuspended in baffled flasks with 0.5 L of M9 medium containing 3 g Na_2_HPO_4_, 1.5 g KH_2_PO_4_, 0.25 g NaCl, 0.5 g ^15^NH_4_Cl, 2 mM MgSO_4_, 100 μM CaCl_2_, 0.4% glucose, 5 mL Gibco MEM vitamins solution (SIGMA) and 0.5 mL trace elements solution (50 mM FeCl_3_, 50 mM ZnSO_4_, 100 mM MnCl_2_, 10 mM CuSO_4_) and 100 µg/mL Kanamycin. Baffled flasks increase oxygenation of the medium during shaking for optimal protein expression. The 0.5 L M9 culture was subsequently incubated at 30°C with shaking until the medium reached an optical density, OD_600_ of 0.8–1.0. The temperature was then reduced to 13°C for one hour before adding the isopropyl β-thiogalactopyranoside (IPTG) to a final concentration of 0.5 mM. The cells were pelleted by centrifugation after incubation at 13°C overnight with shaking at 200 rpm.

*Tr*Bgl2 and the mutant E367Q *Tr*Bgl2 were purified in an analogous manner to that previously described by Jeng et al. [11].

#### ^15^N, ^13^C, ^2^H and IVL methyl groups specifically labelled *Tr*Bgl2 protein

^15^N, ^13^C, ^2^H and IVL methyl groups specifically labelled *Tr*Bgl2 protein was expressed and purified in an analogous manner to that previously described by Makraki (BMRB 50158) [15].

### Enzyme activity assay

A fluorescent kinetic assay was performed for library screening of *Tr*Bgl2 using 500 µM 4-methylumbelliferyl-β-d-glucopyranoside (MUG) in a total volume of 150 µL per well. Compounds dissolved in DMSO were added to each well to yield a final concentration from 1 to 2 mM. The reactions were performed in 160 mM phosphate–citrate buffer in pH 6.0, initiated by adding 20 µL of the enzyme to give a final concentration of 100 nM and were assayed at 40°C. The fluorescent 4-methylumbelliferyl release was recorded continuously at 355 nm excitation and 460 nm emission for 20 s using a BMG Labtech POLARstar OPTIMA plate reader. Control experiments were performed to evaluate the possibility of interference from the intrinsic fluorescence of compounds in the library by repeating the assay in the absence of the enzyme.

AC_50_ measurements of fragment activators were performed using a final concentration of 100 nM protein and 500 µM MUG with various concentrations of compounds depending on the solubility limits of each compound in a total volume of 150 µL per well. Assays were carried out in a 96 well plate (Black Nunc F96 MicroWell Plates) at 40 and 45°C for *Tr*Bgl2 and *Cc*BglA, respectively, in 160 mM phosphate–citrate buffer in pH 6.0 and 2.5% DMSO. Reactions were initiated by the addition of 20 µL of protein to a final concentration of 100 nM, followed by 5 s of shaking. The fluorescent 4-methylumbelliferyl release was recorded continuously at 355 nm excitation and 460 nm emission for 20 s using a BMG Labtech POLARstar OPTIMA plate reader. Rates were calculated using GraphPad Prism 5.0 as the slope of the curve of the collected data. Corrections were made to the detected gradients to adjust for the inner filter effect of MUG and convert the gradients into units of µmol min^−1^ according to a standard curve for MUG. The initial slopes were fitted to sigmoidal dose-response curves using GraphPad Prism 5.0 and the AC_50_ values extracted. Inhibition of *Tr*Bgl2 by isofagomine (IC_50_) was determined in similar manner to the AC_50_ values.

Apparent *K*_M_ and *k*_cat_ values were calculated from a Michaelis–Menten curve fit to 9-point substrate titrations at a constant concentration of compounds. The covariation experiments used the same range of substrate concentrations across an activator and inhibitor concentration range. The data obtained from these experiments was fit to the nonessential activator and mixed-model inhibition equation, respectively.

### Thermal shift assay (TSA)

Thermal shift screens were performed in 96-well polypropylene PCR plates (Agilent 401334) with a final volume 25 µL/well. Well components were E367Q *Tr*Bgl2 (300 nM), SYPRO orange dye (8X), DMSO (5.2%), substrate, MUG (4 mM) and compound at 4 mM in the same buffer as used for the enzymatic assays. Melting data were collected using an Agilent Stratagene Mx3005P rtPCR machine ramping from 25 to 95°C at 30 s per degree. Data were analysed with a combination of the Agilent MxPro software, MTS [16], JTSA (http://paulsbond.co.uk/jtsa) and the DSF analysis spreadsheet in Excel [17].

### NMR spectroscopy

All NMR experiments were collected at 298 K on a Bruker Avance Neo 700 MHZ spectrometer equipped with a triple-resonance cryogenic probe. Data were processed using Topspin 3.2 (Bruker) and analysed with Sparky [18].

#### STD NMR

The samples contained 20 µM unlabelled *Tr*Bgl2, fragments at a concentration of 500 µM per compound and 100 µM sodium trimethylsilylpropanesulfonate (DSS) in 50 mM phosphate buffer containing 25 mM NaCl and 5% D_2_O at pH 6.0. The experiments were recorded by using a Bruker standard pulse sequence (stddiffesgp.3 [19]). The on-resonance frequency was set to 0.25 ppm and off-resonance irradiation was applied at 40 ppm. STD NMR spectra were collected with a total of 16 scans (NS), 5 s delay between the scans (d1), a saturation time of 4 s (d20) and 1.5 s acquisition time (AQ).

#### NMR titrations

For backbone chemical shift perturbations (CSPs), each sample was prepared with 120 µM ^15^N isotope-labelled *Tr*Bgl2 or E367Q *Tr*Bgl2, 8 mM or 16 mM of compound **8** or **10**, 5 mM of MUG substrate and 100 µM DSS in 50 mM TRIS buffer containing 100 mM NaCl and 10% D_2_O at pH 8.0. The final DMSO concentration was usually not above 3.8%(v/v). ^1^H-^15^N TROSY spectra of the enzyme with DMSO in the buffer were collected as controls. ^1^H-^15^N TROSY spectra were collected by using a Bruker standard pulse sequence (trosyetf3gpsi [20,21]) with 128 scans (NS), 1.5 s delay between the scans (d1), 35 ppm spectra width (SW), 256-time domain data size (TD) and 117 ppm irradiation carrier frequency offset (O1P).

For IVL methyl group CSPs, each sample was prepared with 250 µM of ^15^N, ^13^C, ^2^H and IVL methyl group specifically labelled *Tr*Bgl2, 8 mM of compound **8**, in 50 mM TRIS buffer containing 100 mM NaCl and 10% D_2_O at pH 8.0. The final DMSO concentration was usually not above 3.8% (v/v). ^1^H-^13^C HSQC spectra of the enzyme with DMSO in the buffer were collected as controls. ^1^H-^13^C HSQC spectra were recorded by using a Bruker standard pulse sequence (hsqcctetgpsp [22]) with 8 scans (NS), 1.5 s delay between the scans (d1), 30 ppm spectra width (SW), 256-time domain data size (TD) and 15 ppm irradiation carrier frequency offset (O1P).

### Protein–ligand docking

The HADDOCK (High Ambiguity Driven DOCKing) Web server [23,24] used 1000 protein structures for initial rigid-body docking solutions. The best 200 solutions based on their intermolecular energy were used for a semi-flexible refinement. After initial docking iterations, a final explicit solvent refinement was performed on all 200 models which then clustered based on root mean square deviation criteria. Analysis of HADDOCK scores was performed to select the best docking solutions. HADDOCK calculations were driven by the available CSPs recorded after binding of compound **8** to *Tr*Bgl2. Three-dimensional (3D) conformations of the substrate, MUG and ligand (**8**) were generated using SMILES [25] as input.

## Results and discussion

### Library screening

**Figure 2.**
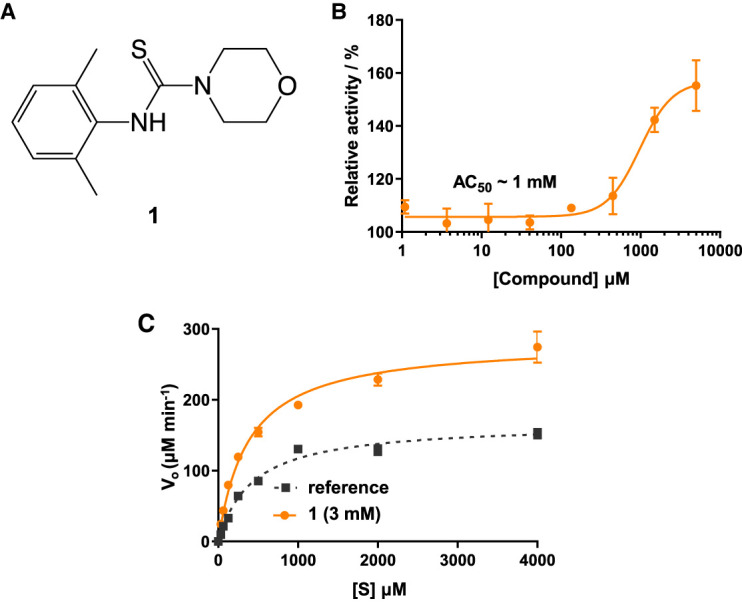
Compound 1 activates *Tr*Bgl2. (**A**) Chemical structure of the initial hit (**1**) identified by fragment screening. (**B**) MUG cleavage assay AC_50_ curve for **1**. Substrate concentration = 500 μM. (**C**) MUG cleavage assay Michaelis–Menten plot for *Tr*Bgl2 in the absence and presence of **1**. [S] = substrate concentration.

A 560 compound (<250 Da) fragment library was screened in an enzymatic assay using 4-MUG as a substrate. In the initial screen, one compound (**1**) enhanced the ability of *Tr*Bgl2 to cleave MUG (Figure 2A). Kinetic characterisation of the hit compound using MUG as substrate showed an activation of *Tr*Bgl2 with A_max_ and AC_50_ value of ∼155% and 1 ± 2 mM, respectively (Figure 2B; A_max_ = maximal activation obtained, AC_50_ = concentration of compound eliciting 50% of maximal activation), whilst Michaelis–Menten analysis of **1** yielded an approximately 2-fold improvement in *k*_cat_*/K*_M_ values (Figure 2C; *k*_cat_ = catalytic rate constant, *K*_M_ = Michaelis constant).

**Figure 3.**
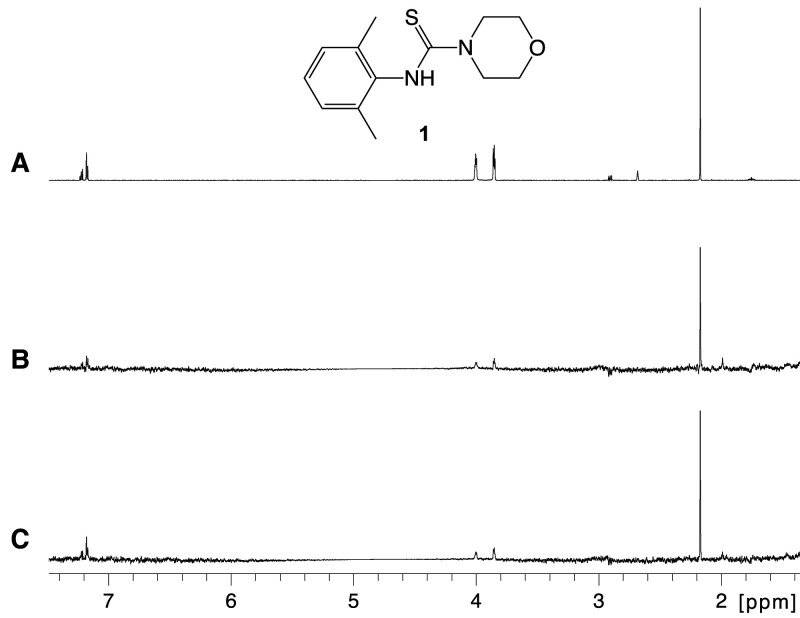
Compound 1 binds to *Tr*Bgl2. (**A**) 1D proton reference spectrum of **1**. (**B**) STD NMR spectrum of **1** (500 µM) in the presence of *Tr*Bgl2 (20 µM) and (**C**) the STD NMR spectrum of the same sample following the addition of isofagomine (**2**) (40 µM).

Ligand-observed NMR spectroscopy was used for binding characterisation of the hit compound (Figure 3). Hit compound (**1**) showed evidence of binding to the enzyme in saturation transfer difference (STD) NMR [19]; binding was also observed in the presence of the known glycoside hydrolase inhibitor isofagomine (**2**) with an IC_50_ value of 0.53 ± 0.12 μM (Supplementary Figure S1). This allowed the categorisation of the hit compound as non-competitive with isofagomine and suggested that binding must occur at an allosteric site.

To improve upon the initial hit, commercially available analogues of compound **1** were identified through an ‘analogue-by-catalogue’ approach. Many these analogues gave an increased activation of *Tr*Bgl2. Replacement of the thiourea group by urea or thio-amide yielded an inactive analogue. Other available compounds that retained the thiourea moiety but had a methyl group in the meta or para (rather than ortho) position were also inactive. As summarised in Table 1, groups other than methyl (such as chloro, methoxy) ortho to the thiourea also yielded active compounds (Supplementary Figure S2). Groups at the ortho position twist the aryl ring out of the plane to avoid steric clashes. This twist of aryl ring might be essential for the activation of *Tr*Bgl2 and could explain why compounds that had a group meta or para instead of ortho appeared to be inactive. Attempts to alter the morpholino moiety were more successful and led to more active compounds. Compound **8** retains the important thiourea and the ortho methyl group generating an activator of *Tr*Bgl2 with significantly improved A_max_ up to 188% in comparison with **1** and the other analogues identified. Compound **8** also had improved solubility (up to 16 mM) compared with other tested compounds and therefore was taken forward for further characterisation. Interestingly, one of the fragment analogues (**10**) that retains the thiourea but does not have ortho substituents appears to be a potent *Tr*Bgl2 inhibitor with an IC_50_ value of 312 ± 32 μΜ (Table 2). Evidence of fragment binding was also observed by STD NMR with a clear signal intensity for both compound **8** (Supplementary Figure S3) and **10** (Supplementary Figure S4) in the presence and absence of isofagomine (**2**). NMR experiments were also attempted to characterise further the orientation of **8** binding; the difference in peak intensities in the STD measurements were not consistent enough for epitope mapping and an NOE experiment was unable to detect any intermolecular NOEs, probably because of the weak affinity of binding.

**Table 1.** Activation of *Tr*Bgl2 by analogues of compound 1

Compound	Chemical structure	AC_50_ (µM)	A_max_ (%)
**3**	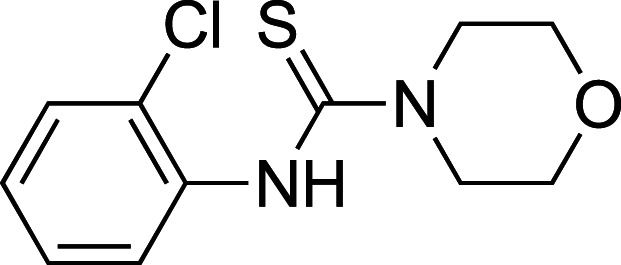	>4000^1^	155^1^
**4**	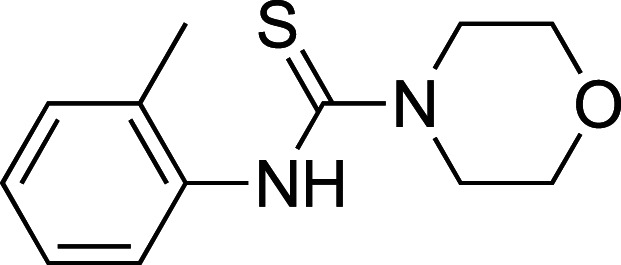	1809±96^1^	139^1^
**5**	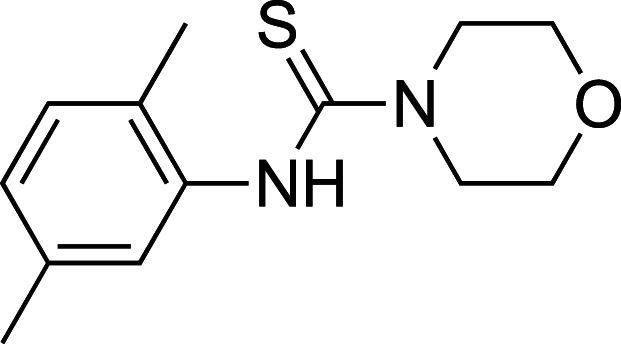	>4000^1^	160^1^
**6**	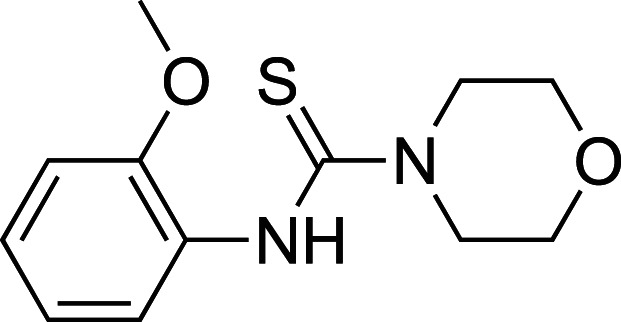	>4000^2^	143^2^
**7**	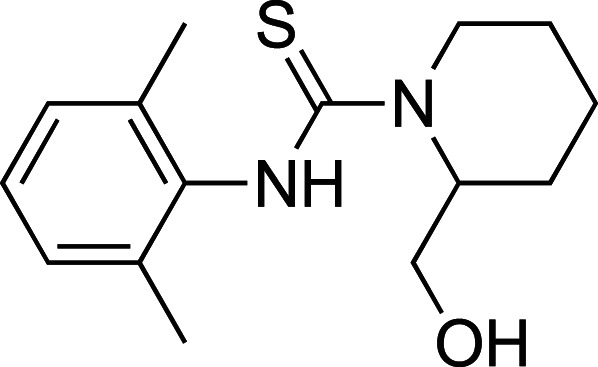	1534 ± 312^2^	161^2^
**8**	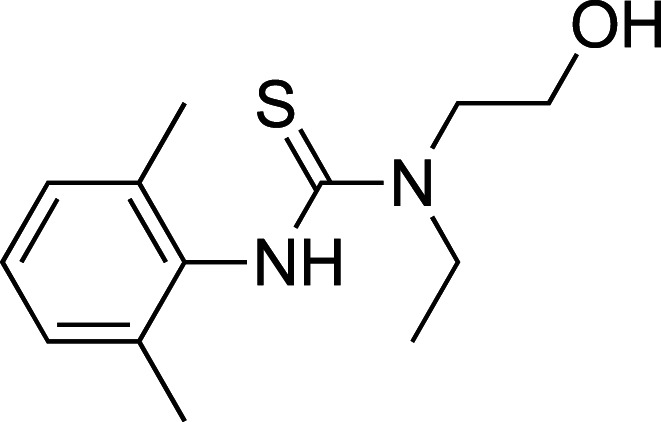	>4000^2^	188^2^
**9**	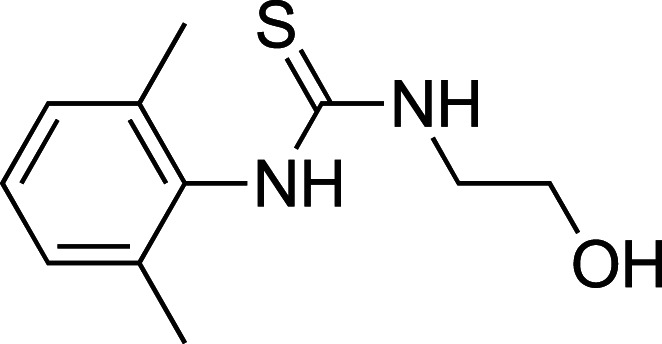	>4000^1^	128^1^

1 Maximum activation obtained with a titration of compound from 30 µM to 3.8 mM (substrate conc. = 500 µM);

2 Maximum activation obtained with a titration of compound from 63 µM to 8 mM (substrate conc. = 500 µM).

**Table 2.** Inhibition of *Tr*Bgl2 by compound 10.

Compound	Chemical structure	IC_50_ (µM)	Maximum inhibition (%)
**10**	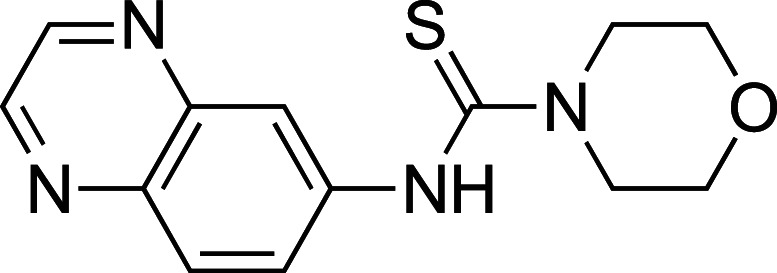	312 ± 32^1^	96^1^

1 Maximum activation obtained with a titration of compound from 63 µM to 8 mM (substrate conc. = 500 µM)

### Kinetic characterisation

**Figure 4.**
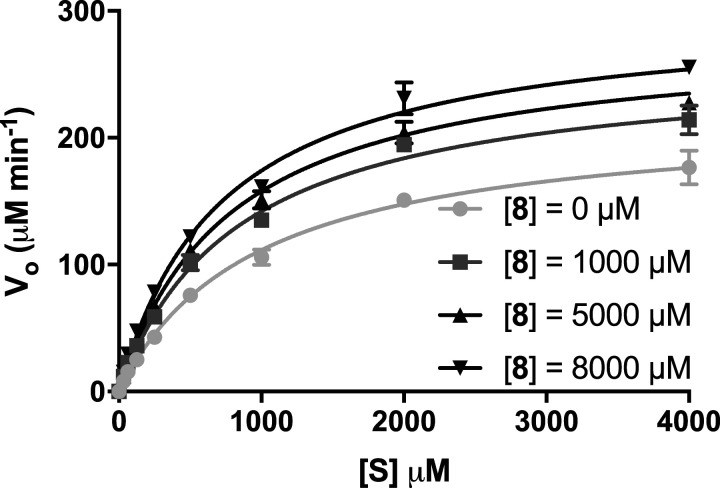
Covariation curve fits to the nonessential activator model for activator 8.

The kinetics of *Tr*Bgl2 activation revealed a nonessential activation model [26], similar to a reversible mixed inhibition model in which the compound displays binding affinity for both the free enzyme and the enzyme–substrate complex. In the nonessential type activation model, the dissociation constant of the substrate and activator are modified in the presence of activator and substrate respectively by the constant *α*. In addition, the *k*_cat_ of the enzyme in the presence of an activator is modified by the constant. Values for the *α* and *β* modifiers were calculated as 0.64 and 1.5, respectively, for activator **8**. The change in substrate binding and catalytic rate constant reflects an approximately 1.5-fold enhancement of substrate affinity and an increase of *k*_cat_ in the presence of activator **8** (Figure 4). Therefore, both values contribute to an enhanced catalytic rate through the enzyme–substrate–activator complex.

Inhibitor **10** has an influence on enzyme kinetics that is consistent with a reversible mixed inhibition model. In this situation, two dissociation constants are defined, one for the binary enzyme–inhibitor complex (*K*_I_) and one for the ternary enzyme–substrate–inhibitor complex (*αk*_I_). The modifier *α* was calculated as 3.65 for inhibitor **10** which indicates an increased dissociation constant for the MUG substrate in the presence of **10** (Supplementary Figure S5).

### Thermal shift assay (TSA)

**Figure 5.**
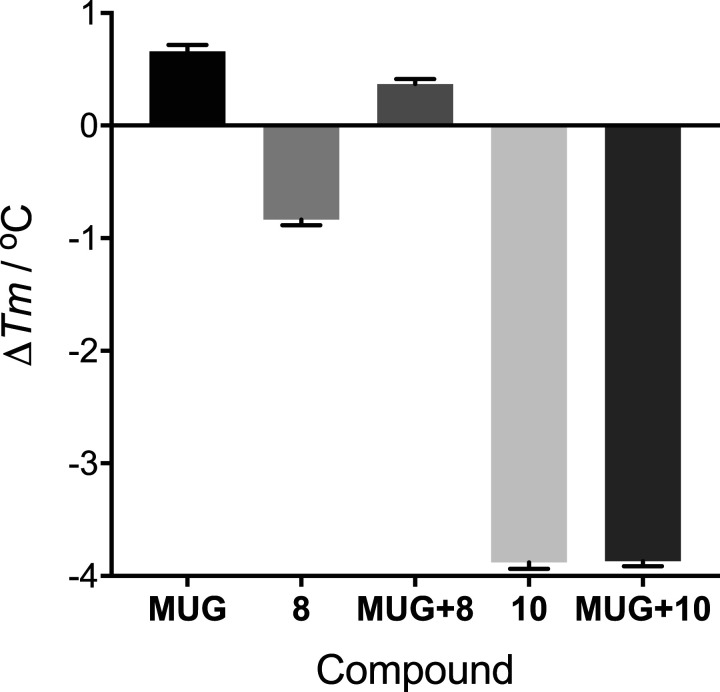
Maximum shift in melting temperature from reference (Δ*T*_m_) for selected compounds and MUG.

To investigate the possible role of the substrate (MUG) in fragment binding, an inactive mutant *Tr*Bgl2 was generated (E367Q *Tr*Bgl2). The influence of fragment binding on E367Q *Tr*Bgl2 stability was assessed by TSA, measuring the melting temperature (*T*_m_) with and without the substrate (MUG) (Figure 5). Both **8** and **10** destabilise apo E367Q *Tr*Bgl2, decreasing the *T*_m_ by ∼1 and 4°C, respectively. In contrast, the destabilising effect of **8** is significantly diminished in the presence of MUG (*T*_m_ increases by ∼1.2°C) whereas the addition of MUG does not affect the significantly large destabilising effect on E367Q *Tr*Bgl2 caused by **10** (*T*_m_ decreases by ∼4°C). This further suggests a contribution of the substrate to fragment binding as the activator affects enzyme activity.

### Selectivity

The bacterial β-glycosidase *Clostridium cellulovorans* (*Cc*BglA) is homologous to the fungal *Tr*Bgl2 sharing 54% sequence similarity (Supplementary Figure S6). None of the fragments activated *Cc*BglA demonstrating that the effect is specific. Interestingly, **10** inhibited both enzymes to a similar degree (Supplementary Figure S7).

### NMR chemical shift perturbation (CSP) experiments

Although the apo structure of *Tr*Bgl2 is known (PDB ID 3AHY) [11], extensive efforts to obtain a crystal structure of **8** bound to either *Tr*Bgl2 or E367Q *Tr*Bgl2 with or without MUG were not successful. We have previously reported the sequential backbone and IVL methyl group partial assignment of the 55 kDa apo *Tr*Bgl2 (BMRB 50158) [15]. Therefore, NMR spectroscopy was used to generate information on the binding site of the activator.

**Figure 6.**
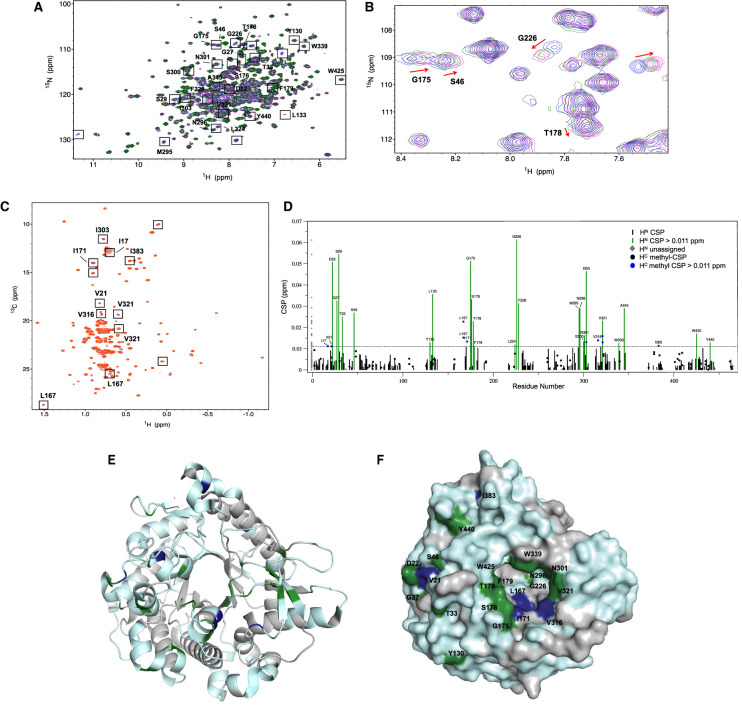
NMR analysis of compound 8 binding to *Tr*Bgl2. (**A**) ^1^H-^15^N TROSY spectrum (700 MHz, 298 K) of apo *Tr*Bgl2 (blue) overlaid with spectra of *Tr*Bgl2 with **8** at 8 mM (green) and 16 mM (magenta). The peaks corresponding to selected backbone amide groups are indicated. (**B**) Expanded panel of ^1^H-^15^N TROSY spectrum. Red arrows indicate the direction of chemical shifts on increasing ligand concentration for selected amino acids. (**C**) CT ^1^H-^13^C HSQC spectrum of an IVL methyl protonated sample (700 MHz, 298 K) of apo *Tr*Bgl2 (purple) overlaid with spectrum of *Tr*Bgl2 with **8** at 8 mM (red). Spectra collected under identical conditions. (**D**) Weighted backbone and methyl CSPs induced by **8**; the dashed line is the experimentally determined significance threshold. (**E**) Assigned backbone and IVL methyl CSPs caused by the presence of **8** mapped onto the crystal structure of *Tr*Bgl2 (PDB ID 3AHY). Assigned backbone and methyl CSPs are coloured green and blue respectively. Unassigned residues are coloured grey. The protein backbone is shown as a ribbon diagram in cyan. (**F**) The above backbone and IVL methyl CSPs mapped on the protein surface, shaded as in (**E**). The figure was created in PyMOL.

First, we investigated the effect of **8** and **10** binding to the protein by recording a 2D ^1^H-^15^N TROSY spectrum in the presence and absence of the compounds. The quality of apo *Tr*Bgl2 ^1^H-^15^N TROSY spectrum obtained, with well-dispersed peaks, is unusually good for such a large protein. Following addition of **8** at 8 and 16 mM some changes in amide chemical shifts occurred across the protein, indicating a specific binding event (Figure 6A,B). Backbone ^1^H-^15^N TROSY CSPs are limited by the fact that most ligand–protein interactions are mediated by sidechains which are distant from the detected backbone amides. Therefore, a sample of deuterated *Tr*Bgl2 with Ile, Val and Leu (IVL) sidechain methyl groups protonated was produced. A constant-time (CT)-[^1^H-^13^C HSQC] spectrum was recorded on this sample in the presence and absence of **8**. Following the addition of **8** at 8 mM, several methyl CSPs were observed reconfirming the binding of **8** to *Tr*Bgl2 (Figure 6C).

CSPs were detected on titration with compound **8** and previously determined assignments [15] allowed some to be mapped onto the known crystal structure of *Tr*Bgl2 (PDB ID 3AHY) (Figure 6E,F). Τhe assignment was not complete [15] but it was sufficient to reveal defined patches of interaction between **8** and *Tr*Bgl2, consistent with a specific binding event at the entrance of the active site. Although most of the CSPs are located at the substrate entrance, residues at the surrounding part of the active site such as D22, G27, T33, S46, Y130 and Y440 are also significantly affected, potentially indicating that conformational changes in the protein are associated with binding of activator **8**. The addition of **10** at 8 mM caused smaller but similar changes as seen for **8** (Supplementary Figure S8). Due to solubility issues ^1^H-^15^N TROSY spectra of **10** at a concentration higher than 8 mM were not recorded.

**Figure 7.**
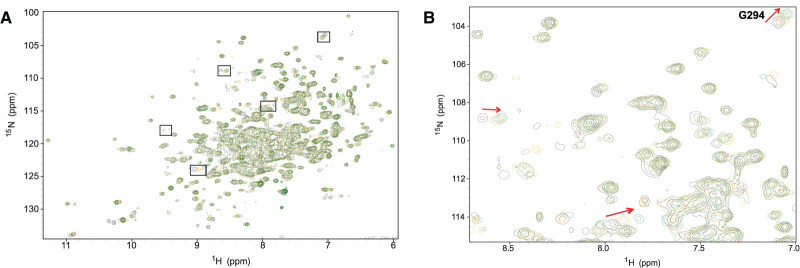
NMR TROSY spectra of 8 and MUG binding to E367Q *Tr*Bgl2. (**A**) ^1^H-^15^N TROSY spectrum (700 MHz, 298 K) of apo E367Q *Tr*Bgl2 (black) overlaid with spectrum of E367Q *Tr*Bgl2 with MUG at 5 mM concentration (orange) and spectrum of E367Q *Tr*Bgl2 bound with both MUG and **8** at 5- and 8-mM concentration, respectively (green). (**B**) Expanded panel of representative portion of the overlaid spectra. Red arrows indicate the direction of chemical shifts. Spectra collected under identical conditions.

The possible role of substrate in fragment binding was further investigated by ^1^H-^15^N TROSY NMR using the inactive mutant, E367Q *Tr*Bgl2. Although the peaks of the spectra are well dispersed, characteristic of a fully folded protein, the resonances were broadened compare to the WT *Tr*Bgl2 spectra. Following the addition of MUG at 5 mM and **8** at 8 mM, the substrate-specific chemical shifts were further enhanced indicating the interaction site remains the same in the ternary complex. Moreover, the magnitude of the substrate-specific chemical shifts is larger in the ternary complex E367Q *Tr*Bgl2–MUG-**8** complex (Figure 7), compared with the binary E367Q *Tr*Bgl2–MUG complex, suggesting that more enzyme is in complex with the substrate. Therefore, considering that the conditions and concentration of MUG were kept identical, these results also indicate higher substrate affinity to the enzyme in the presence of **8**. On the other hand, following the addition of **10** at 4 mM, the substrate-specific chemical shifts were degraded which suggests lower substrate affinity to the enzyme in the presence of **10** (Supplementary Figure S9). Both observations are in full agreement with the kinetic experiments.

### *Tr*Bgl2–8-MUG complex structure

The crystal structure of the closely related *Nk*Bgl–E193D in complex with *p*NPG was used to generate models of the *Tr*Bgl2–MUG and *Tr*Bgl2–**8**-MUG complexes (PDB ID 3IA0) [11] using the program HADDOCK [23,24]. After binding of the substrate (*p*NPG) to the inactive *Nk*Bgl–E193D, significant changes in the structure occur at the side chains of residues Glu/Asp193, Asp253, Trp374, Trp444, Asn449 and Glu451 [11]. The substrate *p*NPG was observed to form direct hydrogen bonds to Gln45, His148, Asn255, Glu402, Trp444, Glu451 and Trp452 of *Nk*Bgl–E193D. A structural alignment suggests that these residues in *Nk*Bgl are equivalent to Gln16, His119, Asp227, Glu367, Trp417, Glu424 and Trp425 of *Tr*Bgl2 (Supplementary Figure S10). The equivalent hydrogen bonding interactions between *Nk*Bgl and *p*NPG can therefore be used as restraints to model the structure of the *Tr*Bgl2–MUG complex (Supplementary Figure S10) using HADDOCK. Asp227, the amino acid that directly interacts with the nitrophenyl part of the substrate, *p*NPG, was excluded. The HADDOCK calculations generated a single cluster (Model 1) which exhibited a HADDOCK score of −60 and a minimum restraint violation energy of 1.1 kcal/mol (Supplementary Figure S11).

**Figure 8.**
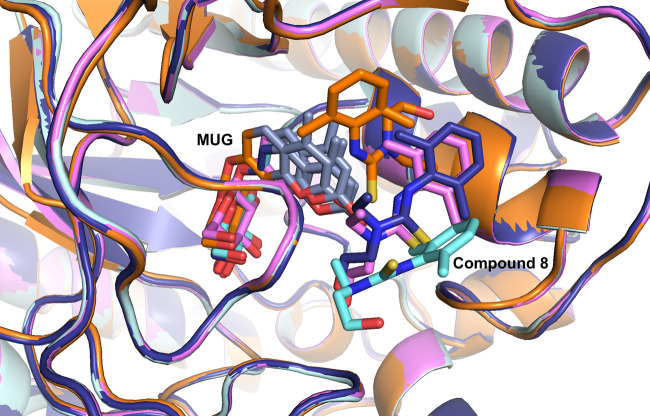
Structural model of compound 8 binding to *Tr*Bgl2. Overlays of the lowest energy HADDOCK model structures of **8** docked with *Tr*Bgl2–MUG complex using the backbone amide and methyl CSPs interpreted to locate at the *Tr*Bgl2's active site. The figure was created in PyMOL.

Next, HADDOCK was also used to generate a model of **8** docked into the *Tr*Bgl2–MUG cluster using model 1, the chemical structure of **8** and the most significant backbone amide and methyl CSPs as ambiguous restraints (assigned as backbone CSPs: G175, S176, T178, F179, G226, M295, N296, N301, W339 and W425; IVL methyl group CPSs: L167, I171, V316 and V321). Because the NMR experiments did not provide sufficient information for epitope mapping or intermolecular NOEs, there is insufficient information to define an orientation for the ligands. However, the HADDOCK calculations generated two clusters indicating the region where the ligand binds. The lowest energy cluster (Model 2) exhibited a HADDOCK score of −19.5 and a minimum restraint violation energy of 35.4 kcal/mol, whereas the next ligand cluster (Model 3) exhibited a HADDOCK score and minimal restraint violation energy of −8.1 and 72.1 kcal/mol, respectively. Both models suggest that the binding site of **8** is at the substrate entrance of *Tr*Bgl2 with a direct contact between activator **8** and MUG (Figure 8). The binding of **8** seems to close the active site entrance, trapping the bound substrate resulting in a more suitable orientation for enzyme activity. Interestingly, the previously identified mutations that lead to enhanced activity of *Tr*Bgl2, such as L167W and T178V [12] are clustered at the same site where the *Tr*Bgl2 activator interacts.

The pattern of mapped CSPs of **10** is quite similar to that of **8** indicating that the two compounds bind in the same binding site (Supplementary Figure S8). It is not clear why **10** is an inhibitor — a possible explanation that is consistent with the kinetic models and the TSA measurements is that **10** significantly destabilises binding of the substrate generating a less productive conformation for enzyme activity. This switch between activation and inhibition with just small modifications of the compound has been observed in other systems, such as for resveratrol-like compounds on sirtuin [27] and in compounds that modulate the interaction between KRas and SOS [28].

## Conclusion

We have used fragment-based methods to identify a selective, small molecule compound that increases the activity of the industrial glycoside hydrolase, *Tr*BglA by 188%. Structure–activity relationship (SAR) of closely related compounds suggest that activation is achieved by substituents inducing a particular conformation on the activators which results in a productive conformation for the enzyme–substrate complex; a closely related compound that was unable to achieve such a conformation was an inhibitor. This work would need to be extended to the covalent linkage of the activators (as previously demonstrated for BtGH84, [29]) to generate enzymes that could feasibly be used for industrial applications. However, it does demonstrate an alternate approach to traditional genetic approaches for generating enzymes with improved activity.

## Open Access

Open access for this article was enabled by the participation of University of York in an all-inclusive Read & Publish pilot with Portland Press and the Biochemical Society under a transformative agreement with JISC.

## Abbreviations

CBH: cellobiohydrolases
CSPs: chemical shift perturbations
FBLD: fragment-based ligand discovery
LC: lignocellulosic
MUG: methylumbelliferyl-β-d-glucopyranoside
PCR: polymerase chain reaction
STD: saturation transfer difference
TSA: thermal shift assay
