# Supplementary material for: Fragment-derived modulators of an industrial β-glucosidase
Source: Biochem J. 2020 Nov 26;477(22):4383–95. doi: 10.1042/BCJ20200507 (PMC7702302; doi:10.1042/BCJ20200507)
Supplement: Supplementary Figures S1-S11 [file BCJ-477-4383-s1.pdf]

Figure S1. A) Chemical structure of isofagomine (**2**). B) Inhibition curve of *Tr*Bgl2 by **2**.

Figure S2. MUG cleavage assay AC_{50} curve for the hit compounds. AC_{50} values obtained with a titration of compounds from A) 30 µM to 3.8 mM and B) 63 µM to 8 mM (substrate concentration = 500 µM). The optimal temperature (40°C) and the optimal pH of *Tr*Bgl2 (pH 6.0) were used in the assay.

Figure S3. A) 1D proton reference spectrum of **8**. B) STD NMR spectrum of **8** (500 µM) in the presence of *Tr*Bgl2 (20 µM) and C) the STD NMR spectrum of the same sample following addition of isofagomine (**2**) (40 µM).

Figure S4. A) 1D proton reference spectrum of **10**. B) STD NMR spectrum of **10** (500 µM) in the presence of *Tr*Bgl2 (20 µM) and C) the STD NMR spectrum of the same sample following addition of isofagomine (**2**) (40 µM).

Figure S5. Covariation curve fits to the reverse mixed inhibition model for inhibitor **10**.

Figure S6. Amino acid sequence alignment of *Tr*Bgl2 and *Cc*BglA enzymes. The secondary structure elements of *Cc*BglA are indicated. Numbering of the principal secondary structure elements underlines the $(\alpha/\beta)_8$-TIM barrel structure; additional secondary elements are indicated by letters. The 3^{10}-helices are indicated by the character η (11). Sequence alignment generated using the T-coffee service (http://tcoffee.crg.cat/apps/tcoffee/index.html). Figure generated with ESPript 3.0 (http://espript.ibcp.fr/ESPript/ESPript/) where identical residues are highlighted in red and similar residues are written with black bold characters and boxed in yellow {Robert, 2014}. The residue numbering is that for *Cc*BglA.

Figure S7. MUG cleavage assay AC_{50} curve for **8** and **10**. AC_{50} values obtained with a titration of compounds from 63 µM to 8 mM (substrate concentration = 500 µM).

The optimal temperature (45°C) and the optimal pH of *Cc*Bgl2 (pH 6.0) were used in the assay.

Figure S8. NMR analysis of compound **10** binding to *Tr*Bgl2. A) ^{1}H-^{15}N TROSY spectrum (700 MHz, 298 K) of apo *Tr*Bgl2 (blue) overlaid with spectrum of *Tr*Bgl2 with **10** at 8 mM (red) concentration. The peaks corresponding to selected backbone amide groups are indicated. Spectra collected under identical conditions. B) Weighted backbone CSPs induced by **10**; the dashed line is the experimentally determined significance threshold. C) Assigned backbone CSPs caused by the presence of **10** mapped onto the crystal structure of *Tr*Bgl2 (PDB ID 3AHY). Assigned backbone CSPs are coloured orange. Unassigned residues are coloured grey. The protein backbone is shown as a ribbon diagram in cyan. D) The above backbone CSPs mapped on the protein surface, shaded as in C. The figure was created in PyMOL.

Figure S9. A) ^{1}H-^{15}N TROSY spectrum (700 MHz, 298 K) of apo E367Q TrBgl2 (black) overlaid with spectrum of E367Q *Tr*Bgl2 with MUG at 5 mM concentration (orange) and spectrum of E367Q TrBgl2 bound with both MUG and **10** at 5- and 4-mM concentration respectively (red). B) Expanded panel of representative portion of the overlaid spectrum. Red arrows indicate the direction of chemical shifts. Spectra collected under identical conditions.

Figure S10. Structural overlay of *Tr*Bgl2 and the homologue *Nk*Bgl-E193D bound to *p*NPG active site is shown as stick diagram. *Tr*Bgl2 (PDB ID 3AHY) is shown in cyan and *Nk*Bgl-E193D bound to *p*NPG (PDB ID 3AI0) is shown in green. *p*NPG is shown as yellow stick diagram. Hydrogen bonds are shown as black dashed lines. The figure was created in PyMOL.

Figure S11. Structural overlay of *Tr*Bgl2 bound to MUG (Model 1) and the homologue *Nk*Bgl-E193D bound to *p*NPG active site is shown as stick diagram. *Tr*Bgl2 bound to MUG (Model 1) is shown in cyan and *Nk*Bgl-E193D bound to *p*NPG (PDB ID 3AI0) is shown in green. MUG is shown in white and *p*NPG is shown in yellow, both as stick diagrams. Hydrogen bonds are shown as black dashed lines. The figure was created in PyMOL.

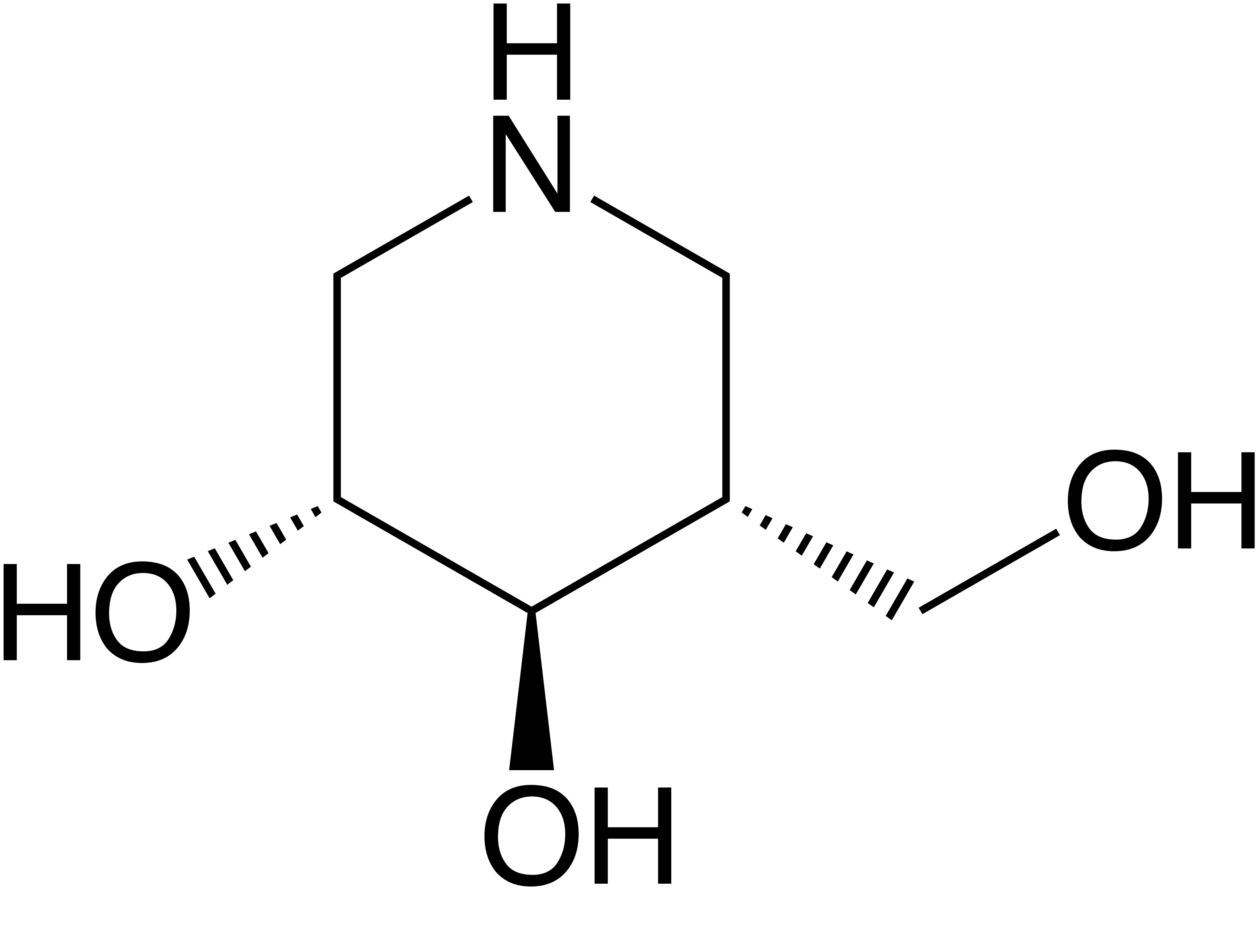

2

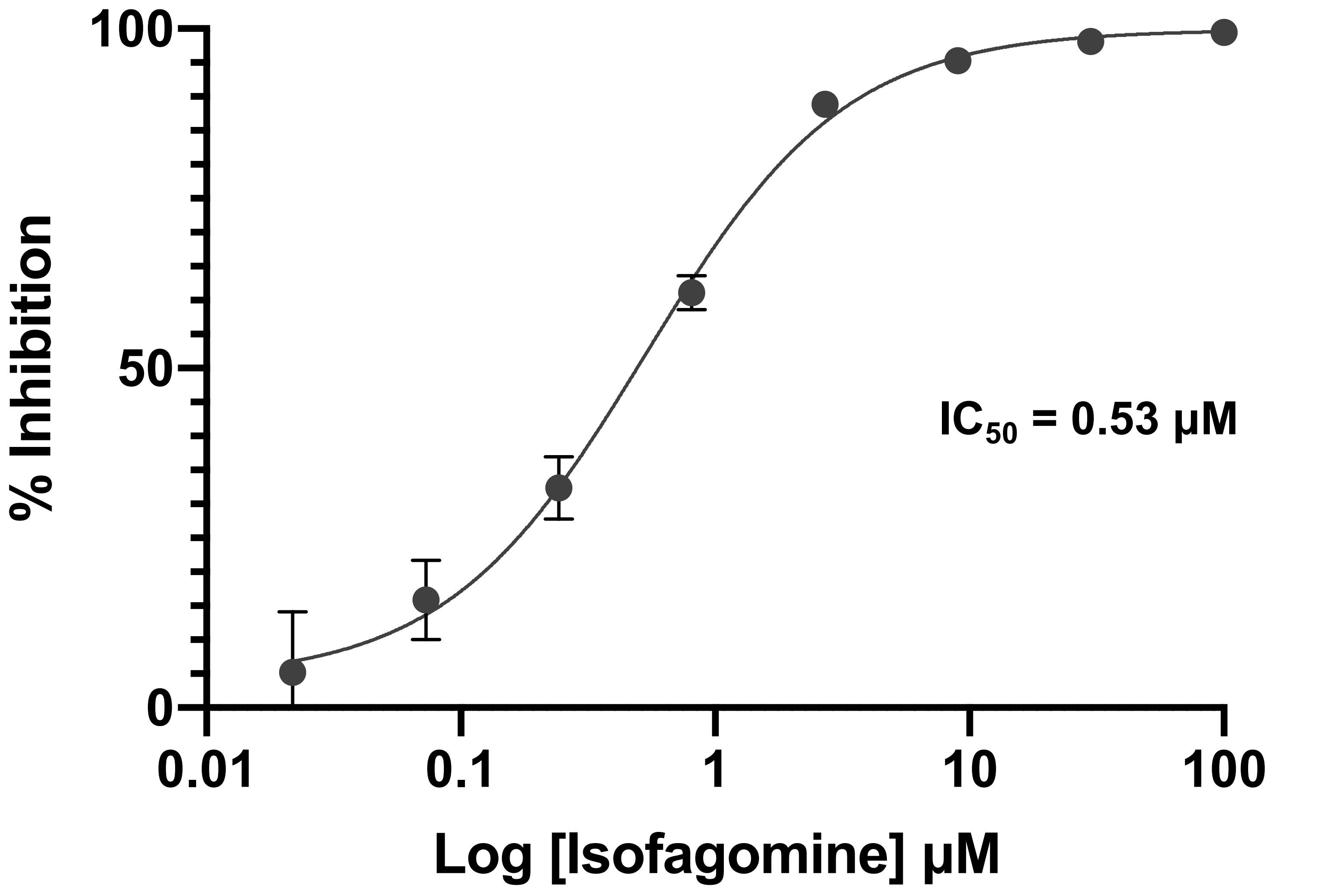

100
50
0
% Inhibition
IC$_{50}$ = 0.53 µM
0.01
0.1
1
10
100
Log [Isofagomine] µM

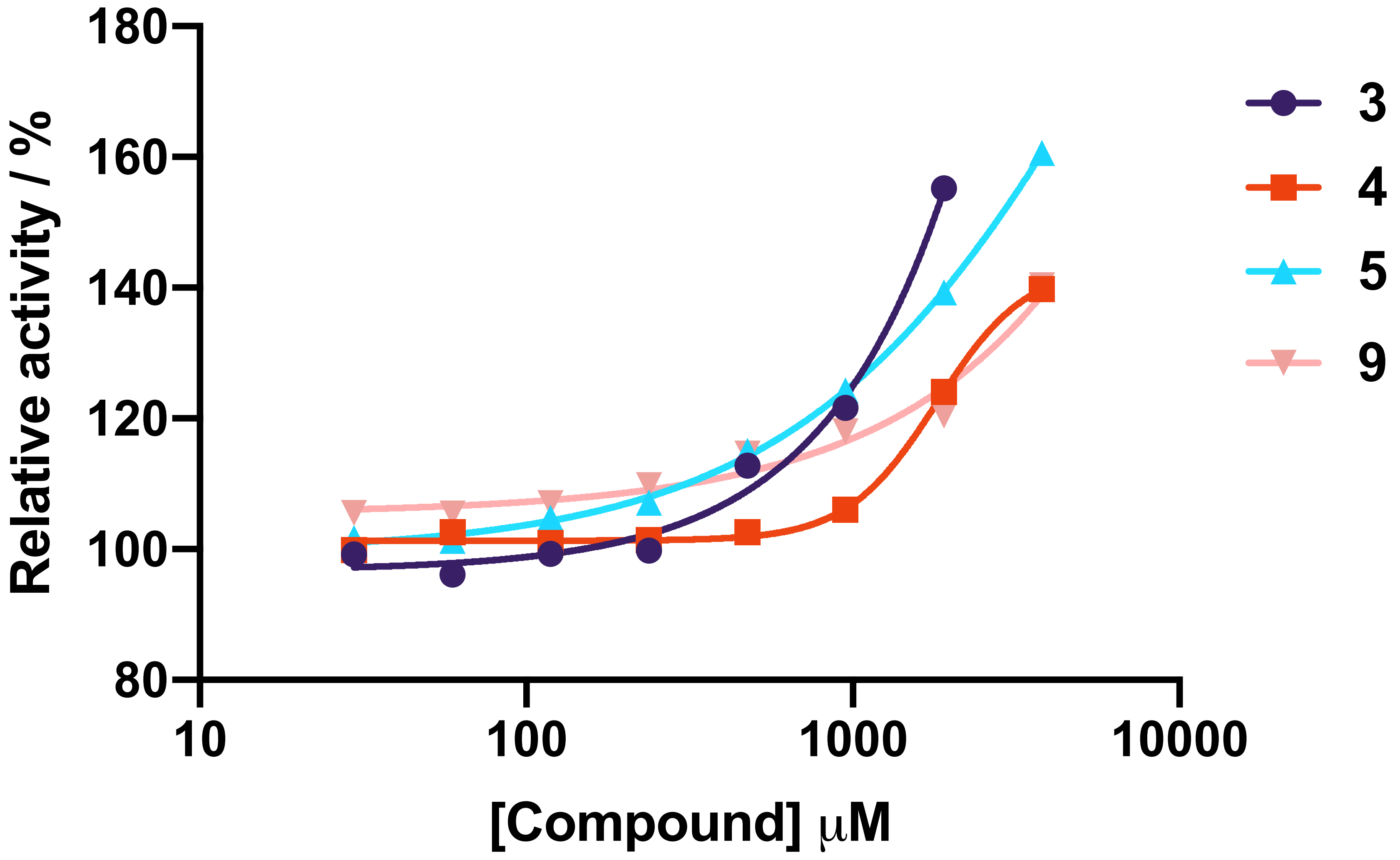

Relative activity / %
180
160
140
120
100
80
10
100
1000
10000
[Compound] μM
3
4
5
9

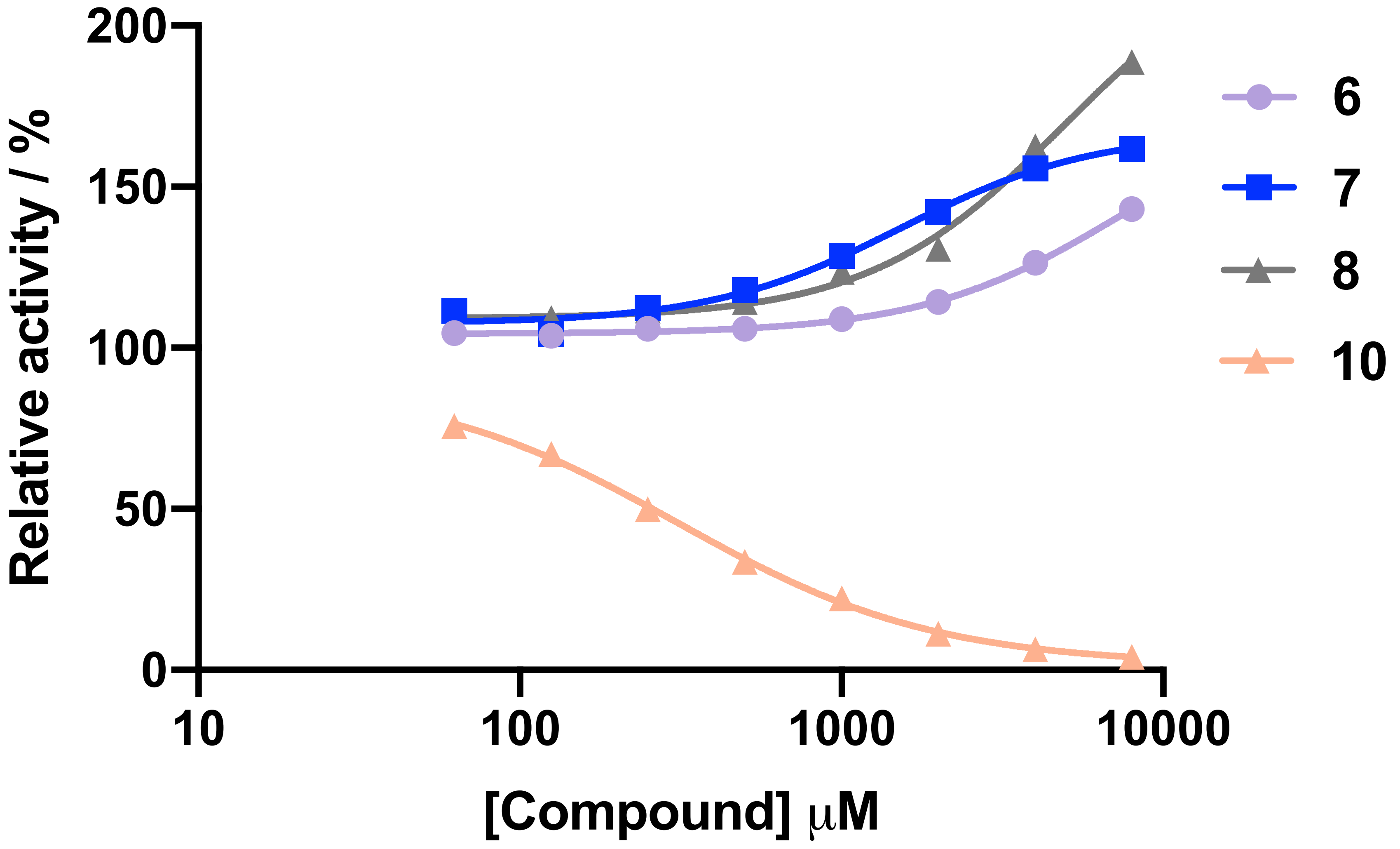

Relative activity / %
200
150
100
50
0
10
100
1000
10000
[Compound] μM
6
7
8
10

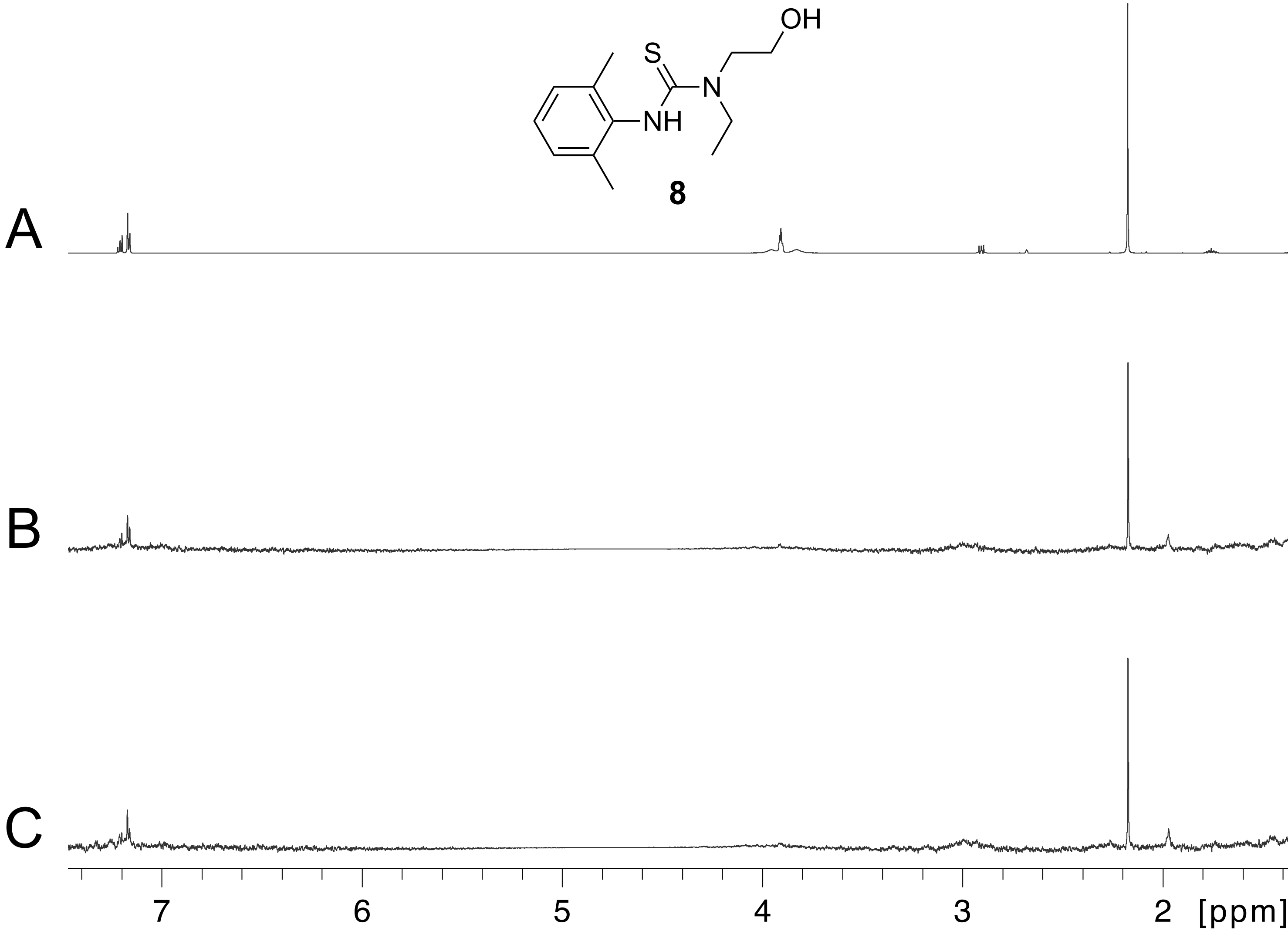

OH
S
N
NH
8
A
B
C
7
6
5
4
3
2
[ppm]

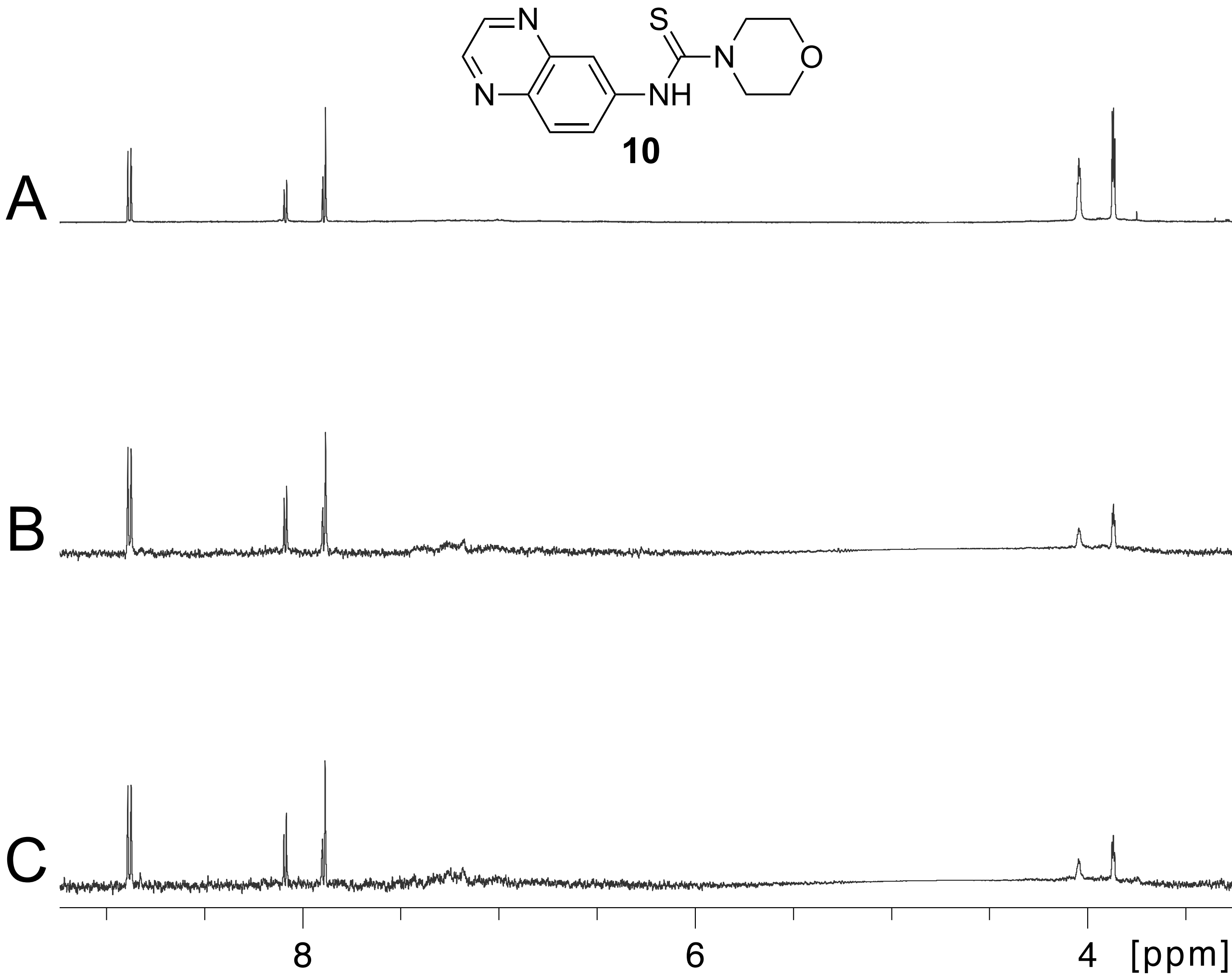

S
N
O
NH
N
10
A
B
C
8
6
4
[ppm]

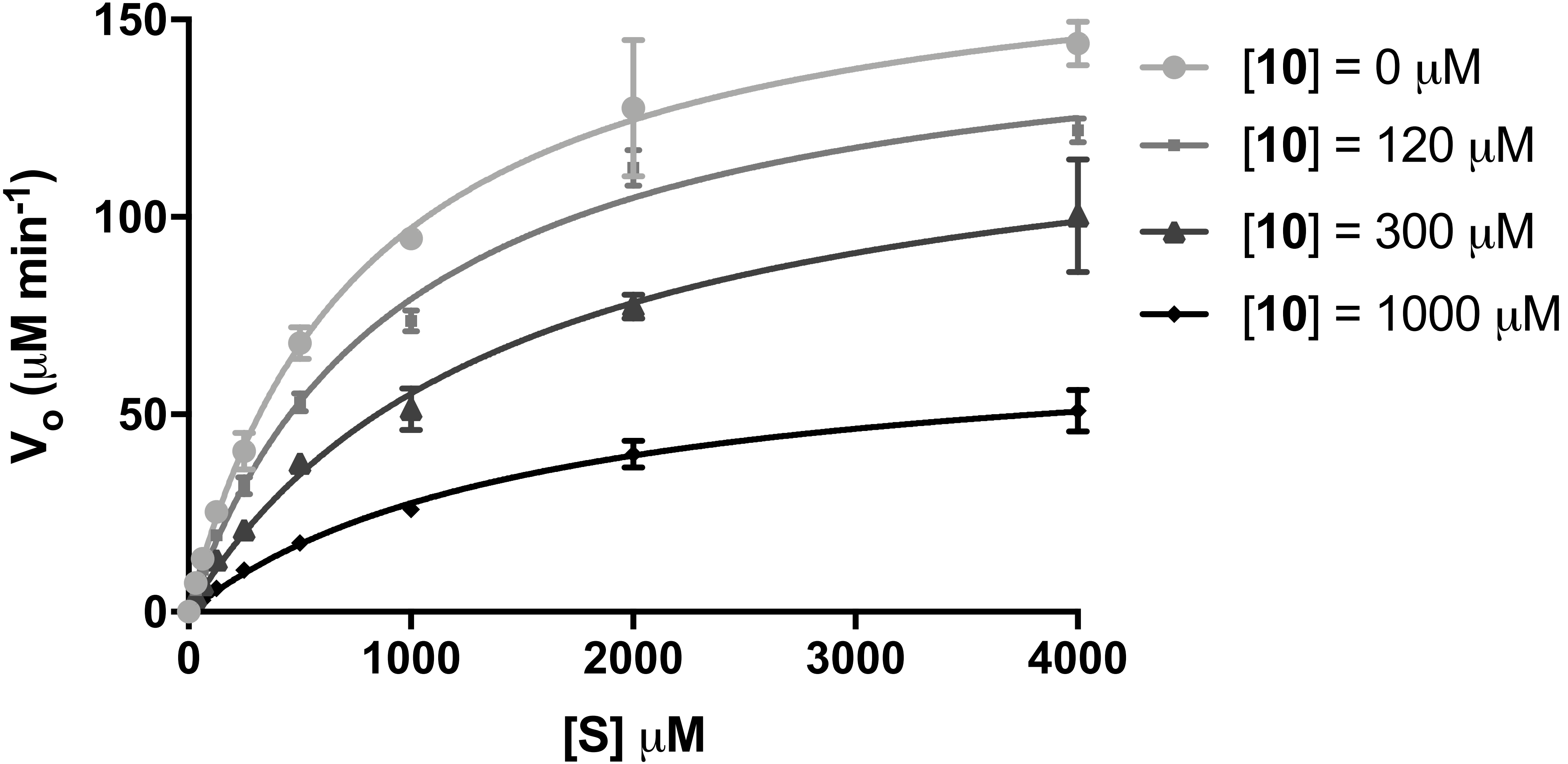

150
100
50
0
0
1000
2000
3000
4000
V_o (μM min^{-1})
[S] μM
[10] = 0 μM
[10] = 120 μM
[10] = 300 μM
[10] = 1000 μM

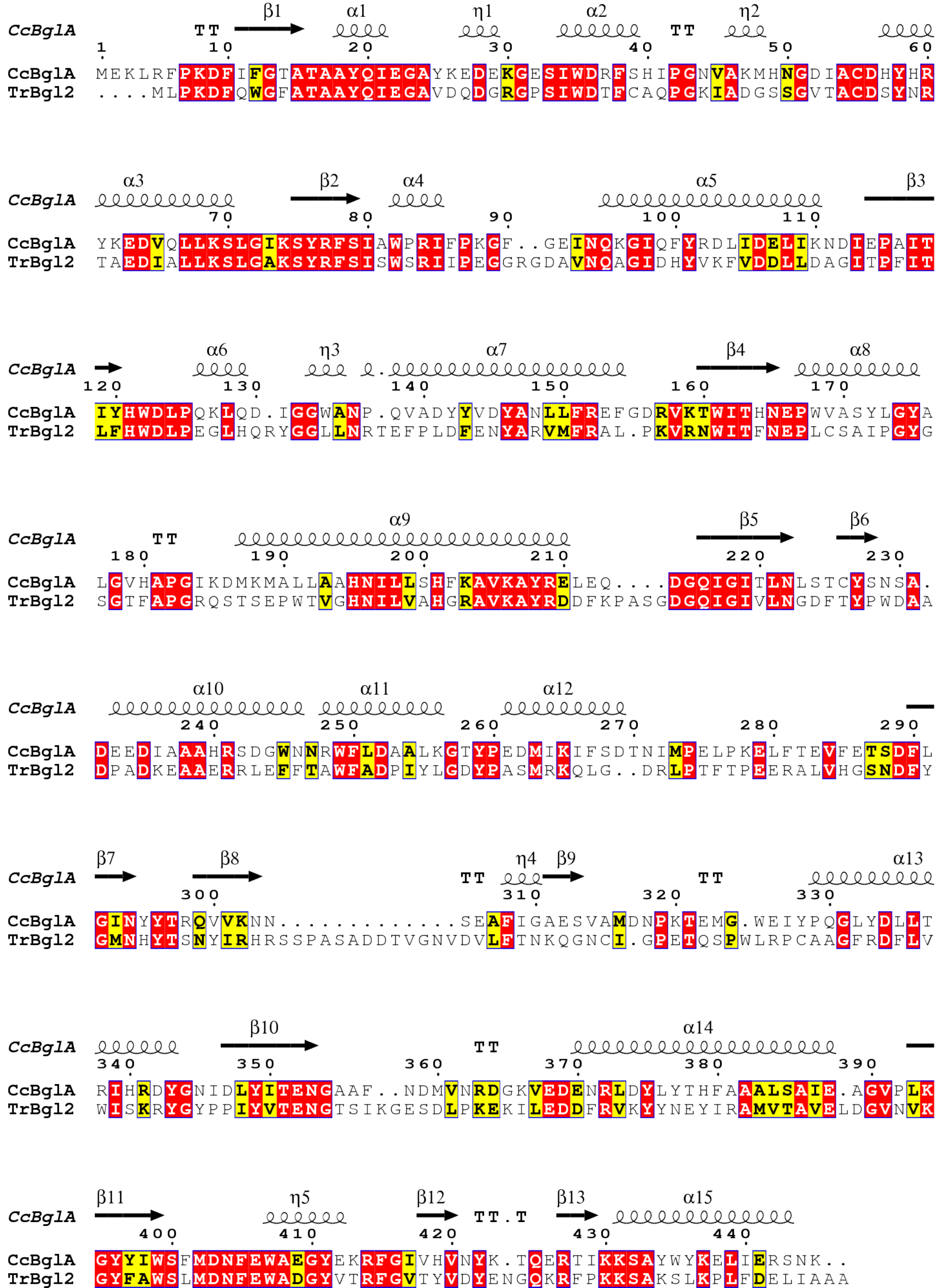

CcBglA
CcBglA MEKLRFPKDFIFGTATAAYQIEGAYKEDEKGESIWDRFSHIPGNVAKMHNGDIACDHYHR
TrBgl2 ....MLPKDFQWGFATAAYQIEGAVDQDGRGPSIWDTFCAQPGKIADGSSGVTACDSYNR
CcBglA YKEDVQLLKSLGIKSYRFSIAWPRIFPKGF..GEINQKGIQFYRDLIDELIKNDIEPAIT
TrBgl2 TAEDIALLKSLGAKSYRFSISWSRIIPEGGRGDAVNQAGIDHYVKFVDDLLDAGITPFIT
CcBglA IYHWDLPQKLQD.IGGWANP.QVADYYVDYANLLFREFGDRVKTWITHNEPWVASYLGYA
TrBgl2 LFHWDLPEGLHQRYGGLLNRTEFPLDFENYARVMFRAL.PKVRNWITFNEPLCSAIPGYG
CcBglA LGVHAPGIKDMKMALLAAHNILLSHFKAVKAYRELEQ....DGQIGITLNLSTCYSNSA.
TrBgl2 SGTFAPGRQSTSEPWTVGHNILVAHGRAVKAYRDDFKPASGDGQIGIVLNGDFTYPWDAA
CcBglA DEEDIAAAHRSDGWNNRWFLDAALKGTYPEDMIKIFSDTNIMPELPKELFTEVFETSDFL
TrBgl2 DPADKEAAERRLEFFTAWFADPIYLGDYPASMRKQLG..DRLPTFTPEERALVHGSNDFY
CcBglA GINYYTRQVVKNN.............SEAFIGAESVAMDNPKTEMG.WEIYPQGLYDLLT
TrBgl2 GMNHYTSNYIRHRSSPASADDTVGNVDVLFTNKQGNCI.GPETQSPWLRPCAAGFRDFLV
CcBglA RIHRDYGNIDLYITENGAAF..NDMVNRDGKVEDENRLDYLYTHFAAALSAIE.AGVPLK
TrBgl2 WISKRYGYPPIYVTENGTSIKGESDLPKEKILEDDFRVKYYNEYIRAMVTAVELDGVNVK
CcBglA GYYIWSFMDNFEWAEGYEKRFGIVHVNYK.TQERTIKKSAYWYKELIERSNK..
TrBgl2 GYFAWSLMDNFEWADGYVTRFGVTYVDYENGQKRFPKKSAKSLKPLFDELIAAA

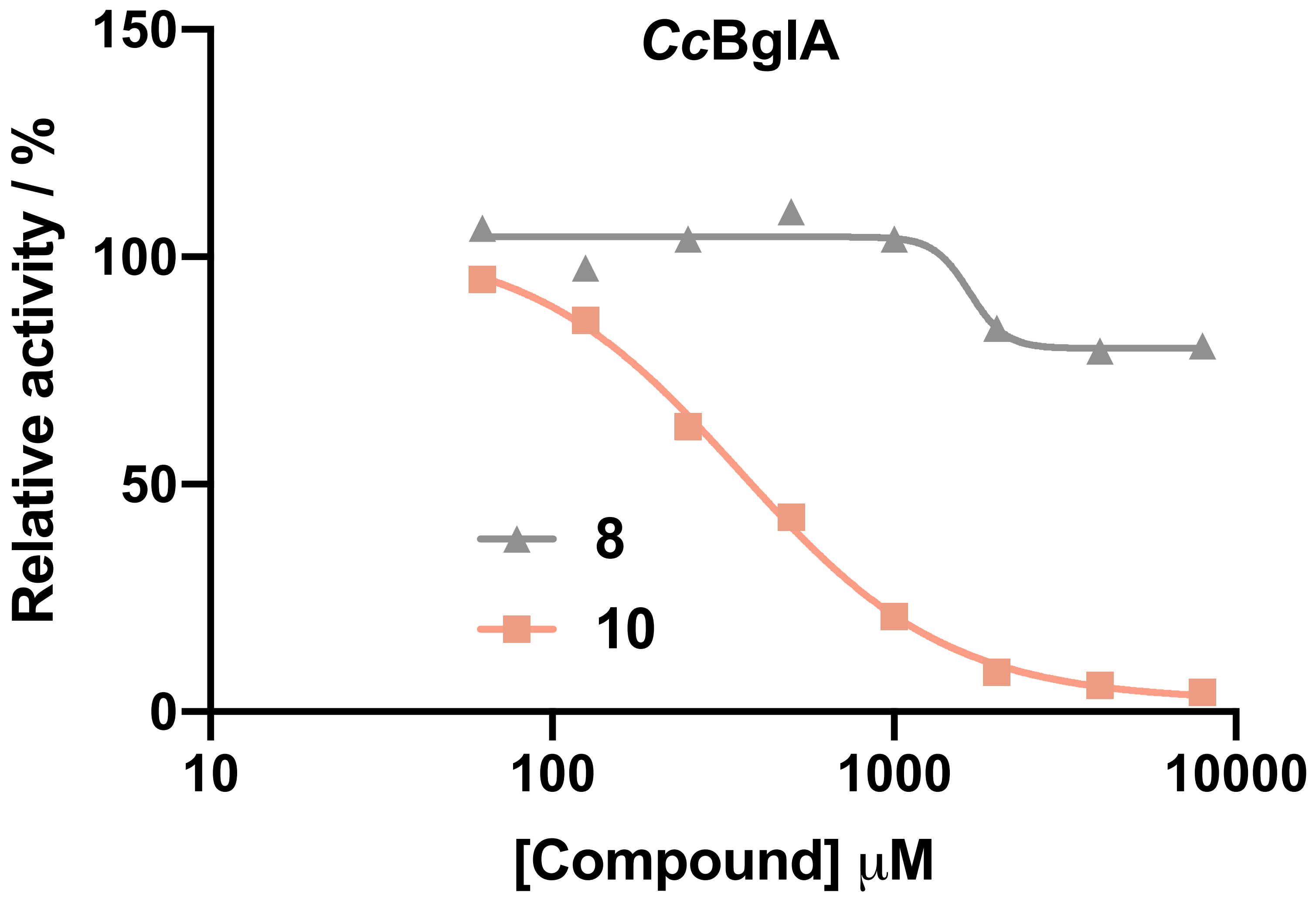

*Cc*BglA
Relative activity / %
150
100
50
0
10
100
1000
10000
[Compound] μM
8
10

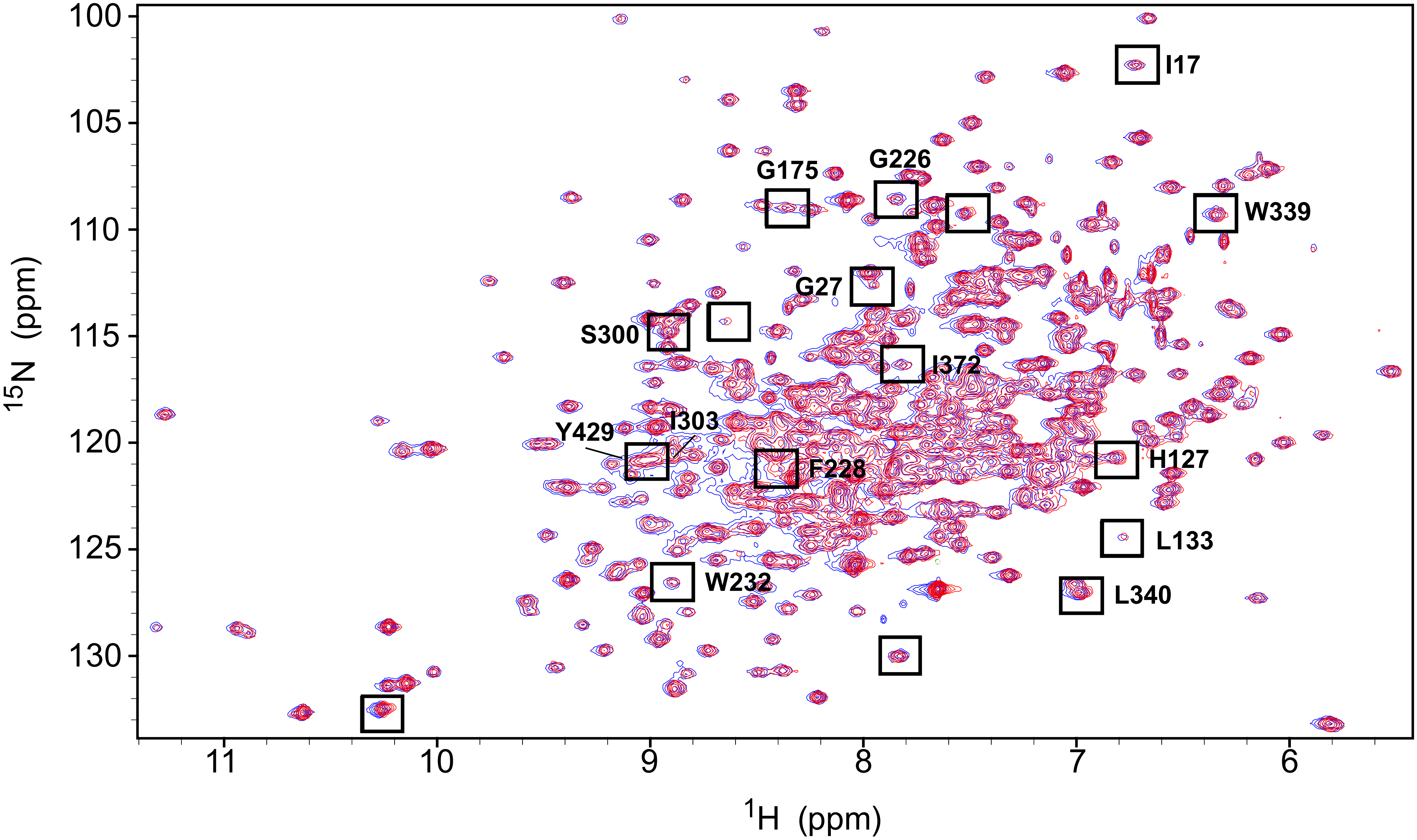

I17
G175
G226
W339
G27
S300
I372
Y429
I303
F228
H127
L133
W232
L340
100
105
110
115
120
125
130
^{15}N (ppm)
11
10
9
8
7
6
^{1}H (ppm)

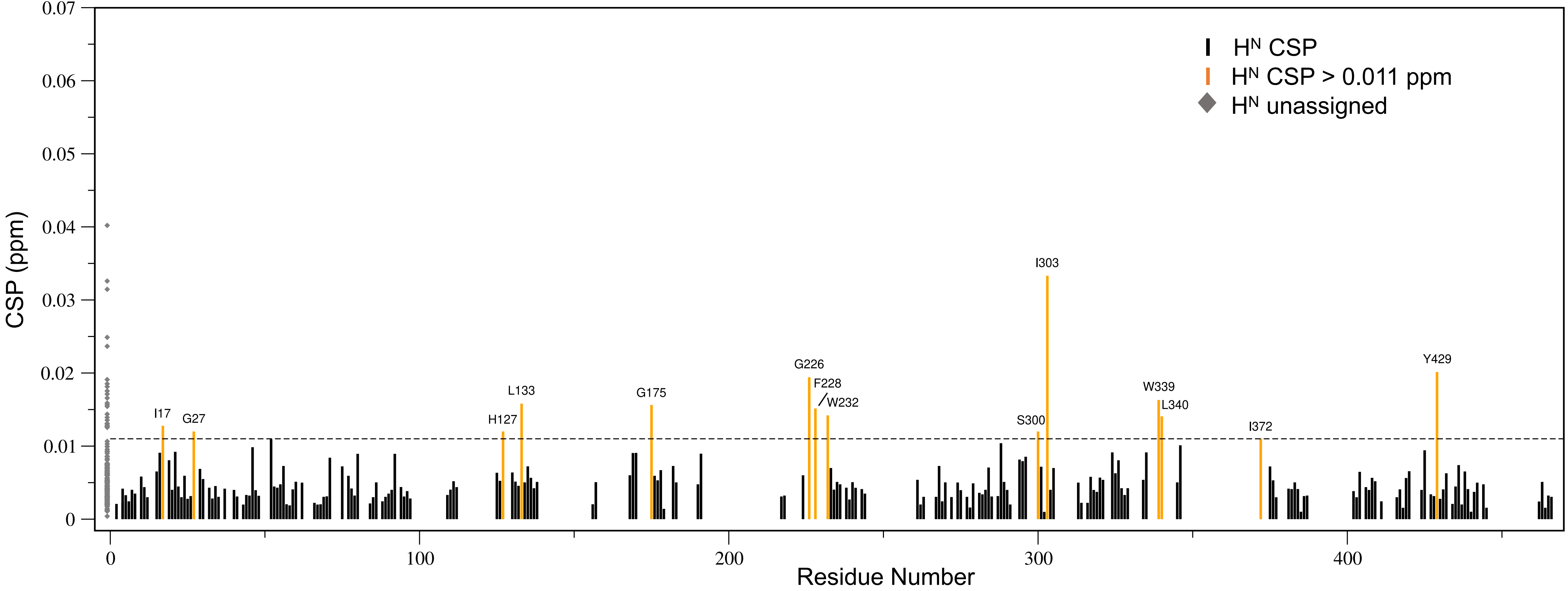

CSP (ppm)
0.07
0.06
0.05
0.04
0.03
0.02
0.01
0
Residue Number
0
100
200
300
400
H^N CSP
H^N CSP > 0.011 ppm
H^N unassigned
I17
G27
H127
L133
G175
G226
F228
W232
S300
I303
W339
L340
I372
Y429

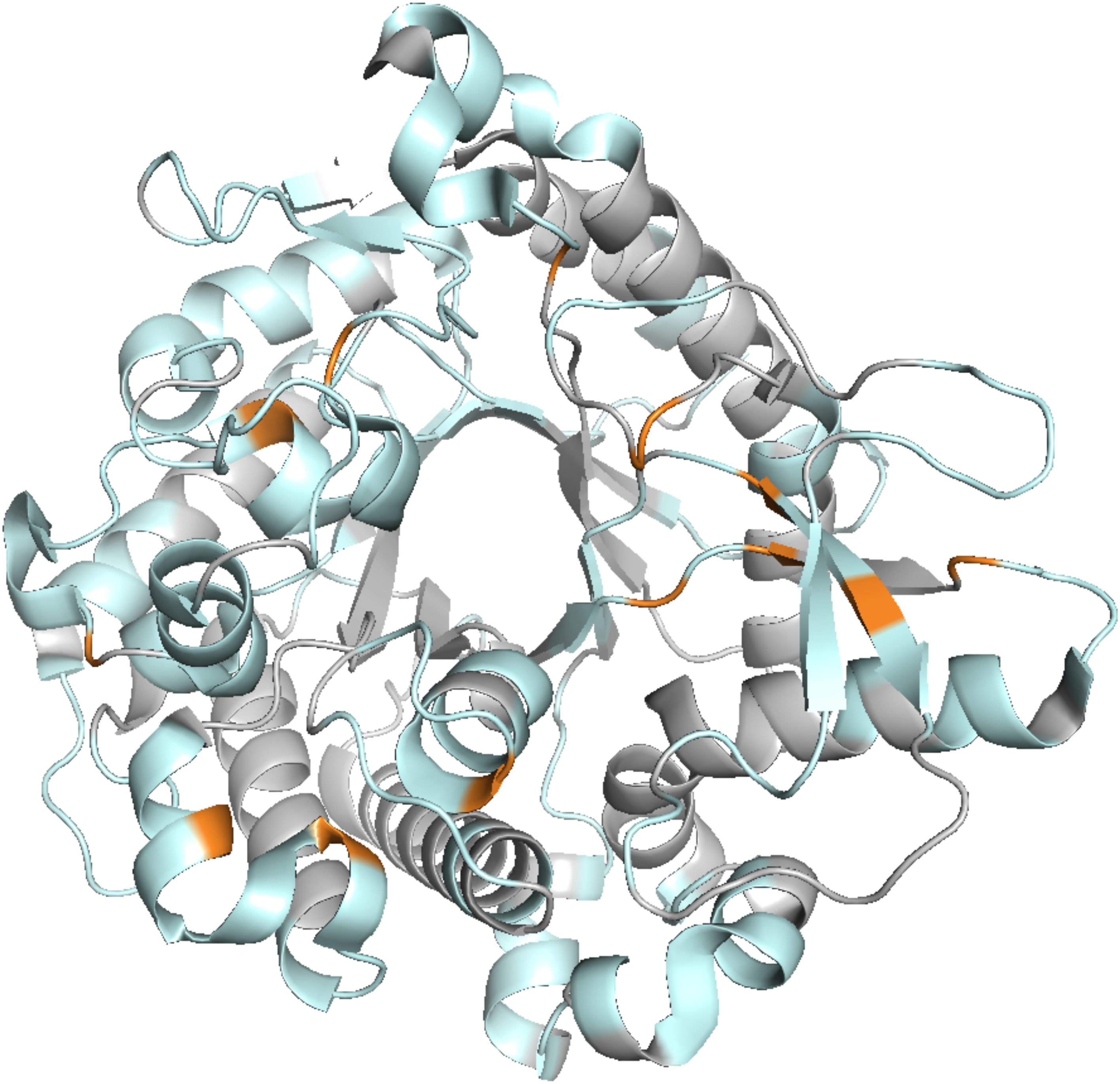

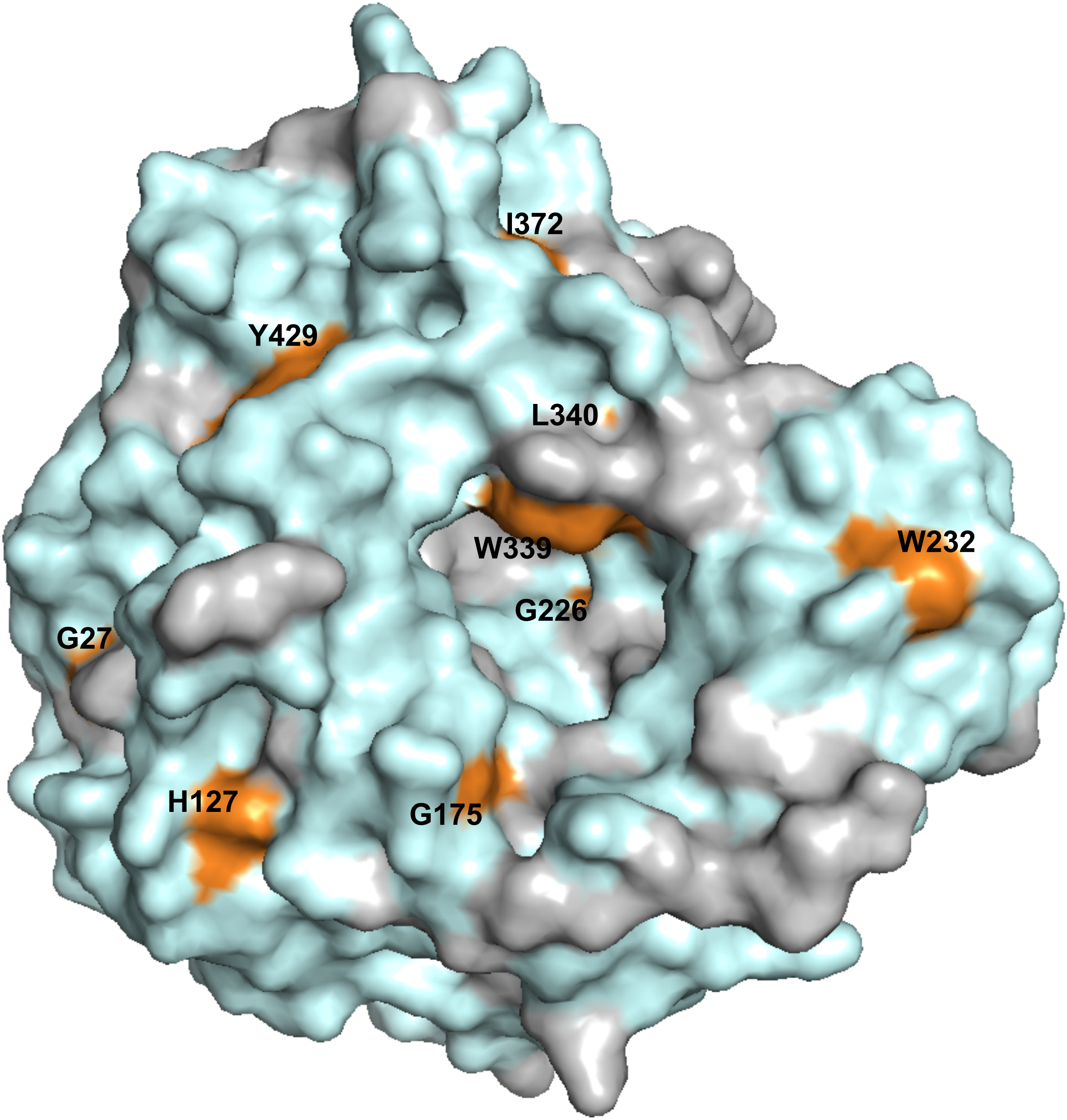
I372
Y429
L340
W339
W232
G226
G27
H127
G175

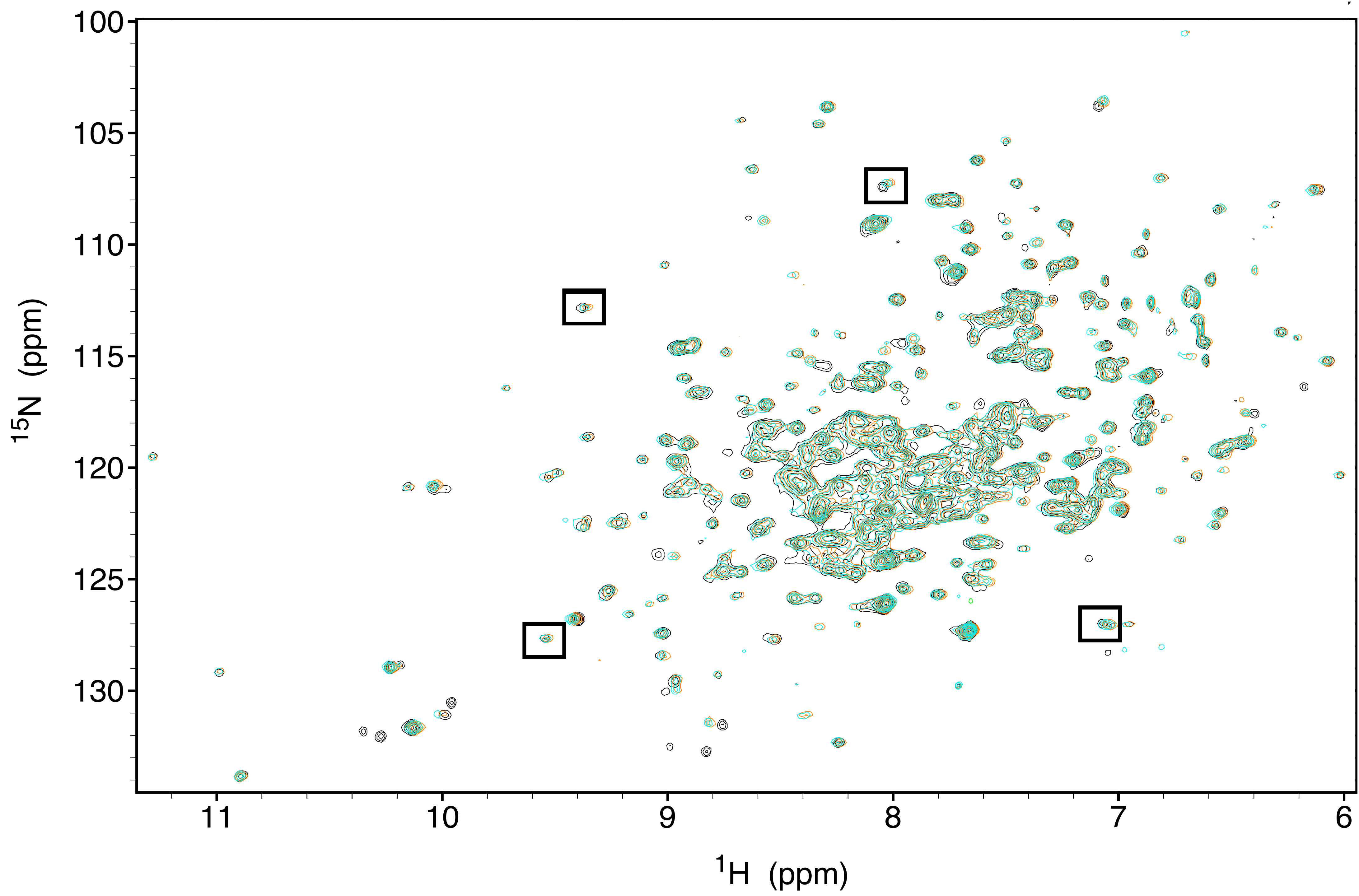

100
105
110
115
120
125
130
^{15}N (ppm)
11
10
9
8
7
6
^{1}H (ppm)

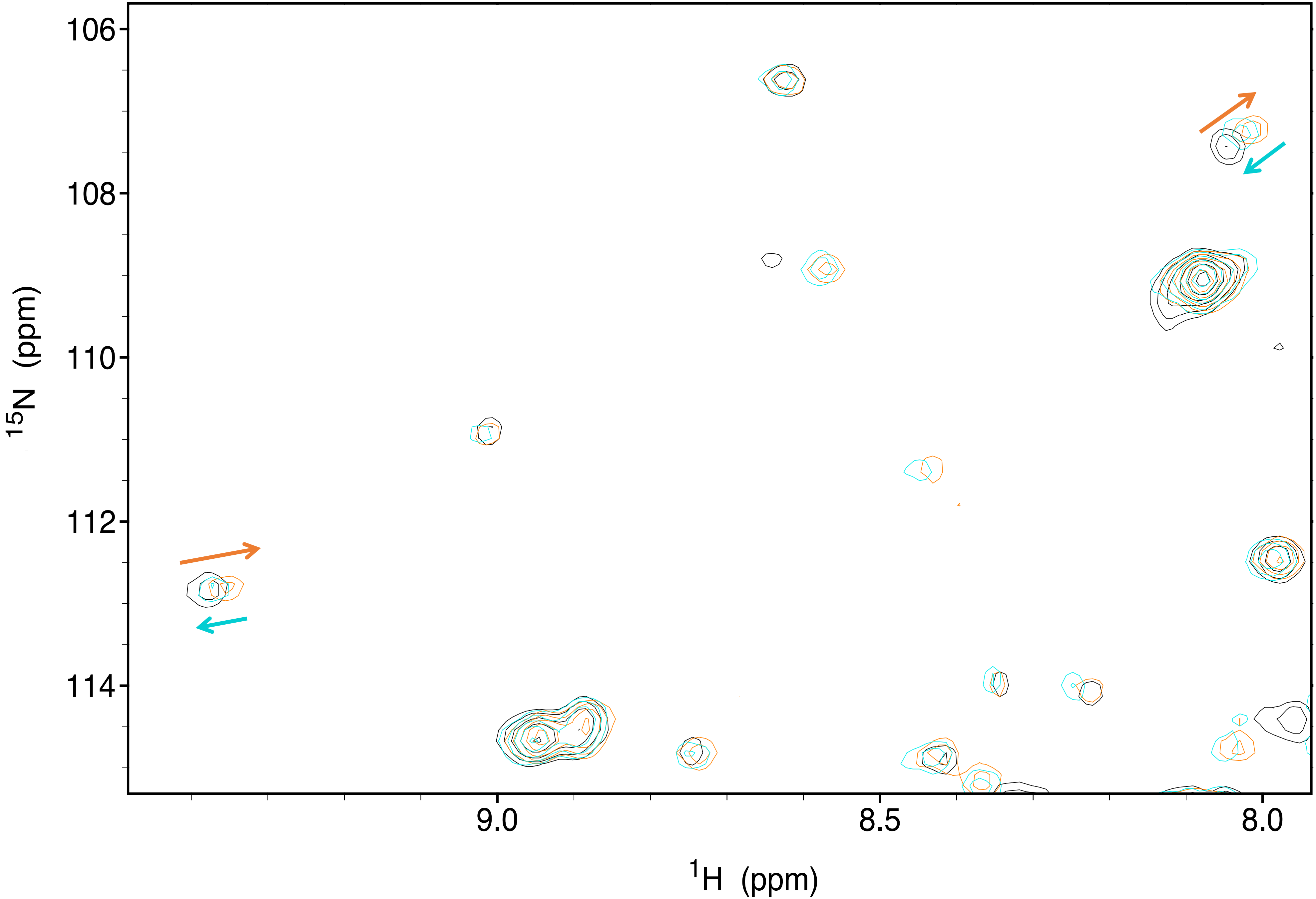

106
108
110
112
114
^{15}N (ppm)
9.0
8.5
8.0
^{1}H (ppm)

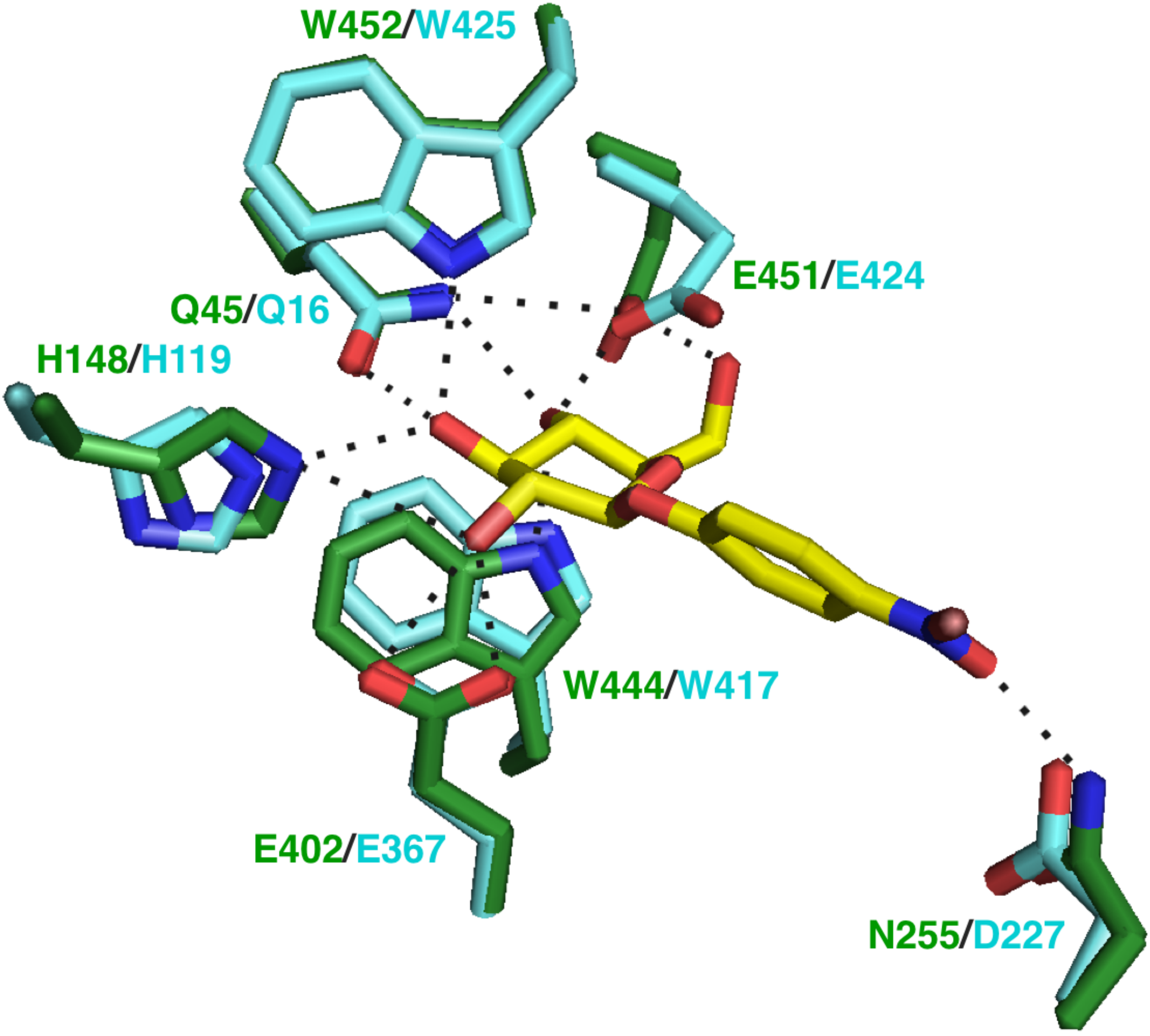
W452/W425
E451/E424
Q45/Q16
H148/H119
W444/W417
E402/E367
N255/D227

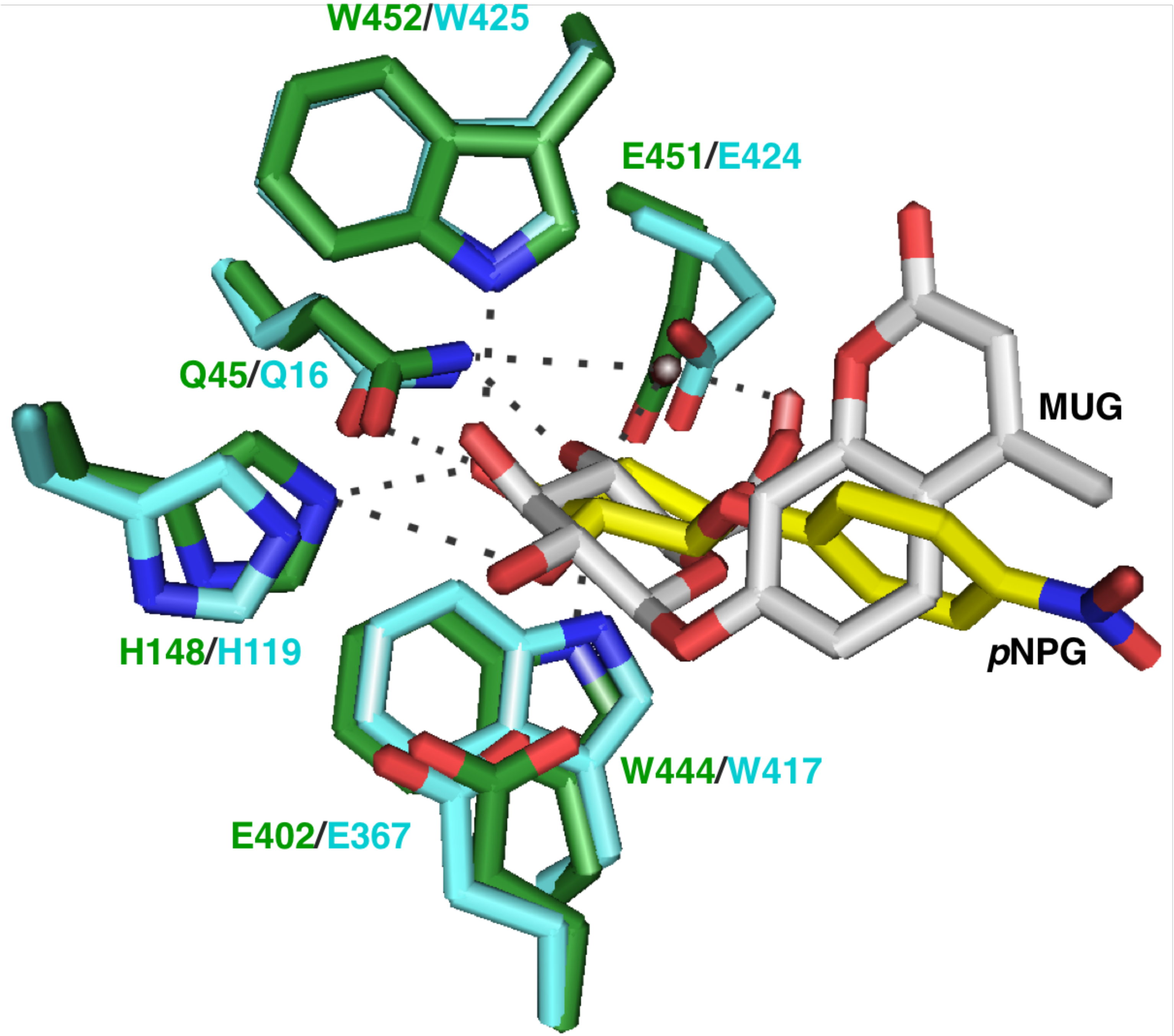
W452/W425
E451/E424
Q45/Q16
MUG
H148/H119
pNPG
W444/W417
E402/E367
